# Short-Term Effects of Primary and Secondary Particulate Matter on Ceramide Metabolism, Pro-Inflammatory Response, and Blood Coagulation

**DOI:** 10.3390/toxics12030225

**Published:** 2024-03-19

**Authors:** Bin Zhang, Hongbing Xu, Xinghou He, Tong Wang, Mengyao Li, Xuyang Shan, Yutong Zhu, Changjie Liu, Qian Zhao, Xiaoming Song, Yele Sun, Lemin Zheng, Wei Huang

**Affiliations:** 1 Department of Occupational and Environmental Health, Peking University School of Public Health, Peking University Institute of Environmental Medicine, Beijing 100191, China; zhangbin2022@bjmu.edu.cn (B.Z.); xuhongbing@bjmu.edu.cn (H.X.); 2111110178@bjmu.edu.cn (X.H.); wtyc112@pku.edu.cn (T.W.); 1710306109@pku.edu.cn (M.L.); shanxuyang@pku.edu.cn (X.S.); zhuyutong@bjmu.edu.cn (Y.Z.); qian-zh@bjmu.edu.cn (Q.Z.); xiaomingsong@bjmu.edu.cn (X.S.); 2 State Key Laboratory of Vascular Homeostasis and Remodeling, Peking University, Beijing 100191, China; liucj1994@126.com; 3 Institute of Cardiovascular Sciences and Institute of Systems Biomedicine, Peking University School of Basic Medical Sciences, Beijing 100191, China; 4 State Key Laboratory of Atmospheric Boundary Layer Physics and Atmospheric Chemistry, Institute of Atmospheric Physics, Chinese Academy of Sciences, Beijing 100191, China; sunyele@mail.iap.ac.cn

**Keywords:** particulate matter, secondary organic aerosols, chemical components, cardiovascular health

## Abstract

Evidence of the precise biological pathway responsible for acute cardiovascular events triggered by particulate matter (PM) exposure from anthropogenic emissions is sparse. We investigated the associations of biomarkers relevant to the pathophysiology of atherothrombosis (ceramide metabolism, pro-inflammatory response, and blood coagulation) with primary and secondary components in particulate matter with aerodynamic diameters less than 2.5 μm (PM_2.5_). A total of 152 healthy participants were followed with four repeated clinical visits between September 2019 and January 2020 in Beijing. Exposure to ambient inorganic aerosols (sulfate, nitrate, ammonium, and chloride), as well as organic aerosols (OA) in PM_2.5_, was measured by a real-time aerosol chemical speciation monitor, and sources of OA were performed by positive matrix factorization. We found significant increases of 101.9–397.9% in ceramide indicators associated with interquartile-range increases in inorganic aerosols and OA prior to 72 h of exposure. Higher levels of organic and inorganic aerosols in PM_2.5_ were associated with increases of 3.1–6.0% in normal T cells regulated upon activation and expressed and secreted relevant to the pro-inflammatory response; increases of 276.9–541.5% were observed in D-dimers relevant to coagulation. Detrimental effects were further observed following OA exposure from fossil fuel combustion. Mediation analyses indicated that ceramide metabolism could mediate the associations of PM_2.5_ components with pro-inflammatory responses. Our findings expand upon the current understanding of potential pathophysiological pathways of cardiovascular events posed by ambient particulates and highlight the importance of reducing primary and secondary PM from anthropogenic combustions.

## 1. Introduction

A report from the World Health Organization (WHO) suggests that 13.7 million deaths could be attributed to air pollution, and nearly 4.2 million deaths were caused by exposure to ambient particulate matter with aerodynamic diameters less than 2.5 μm (PM_2.5_) [1]. Although the precise biological pathway remains to be elucidated, ambient PM_2.5_ exposure has been associated with increased risks of atherothrombotic events [2]. Notably, adverse health effects of PM_2.5_ are closely intertwined with its chemical components and sources [3]. Studies have shown that secondary particulate matter (SPM) components, including sulfate, nitrate, ammonium, and secondary organics, can account for a large proportion of PM_2.5_ mass concentrations [4]. Emerging epidemiological evidence has reported positive associations between short-term exposure to sulfate and nitrate in PM_2.5_ and its sources and an increased risk of cardiovascular mortality [5,6]. Evidence from a population-based investigation has also indicated that the associations of per unit mass of PM_2.5_ secondary organic aerosols (SOA) exposure with county-level cardiorespiratory death risks are stronger than that of PM_2.5_ exposure [7]. A better understanding of the health impacts regarding biological interlinks and pre-clinical responses of primary and secondary PM in humans is critical for cardiovascular disease management and effective control of air pollution emissions.

Recent studies have indicated that ceramides, the base building block for complex sphingolipids, could initiate responses that make cells deal with the lipid burden, which thereby might contribute to the pathophysiology of atherosclerosis, including increased systemic inflammation and coagulation activation [8,9]. Significant increases in levels of ceramides in lung cancer cells were observed in association with a 1-day average of PM air pollution exposure in an in vitro study [10]. Productions of ceramide in mouse lung cells were observed following 7-day diesel exhaust exposure in an in vivo study [11]. Studies have further revealed that ceramides as pro-inflammatory agents could enhance neutrophilic activation, infiltration, and apoptosis [12,13,14]. Higher levels of circulating ceramides were closely correlated with elevations of inflammatory cytokines, such as monocyte chemoattractant protein-1 (MCP-1) [15]. Moreover, ceramides have been a novel element in the regulation of coagulation [16,17], and the changes in ceramides were associated with altered blood coagulation function among participants with high risk of cardiovascular diseases [18]. To date, several studies have suggested that ceramides may play an important role in air pollution-associated atherosclerotic progressions, but evidence in humans is limited [19]. Furthermore, whether the changes in ceramides and inflammatory responses could mediate the primary and secondary PM-associated coagulation activation remains poorly understood and merits investigation.

We have previously shown that SPM (e.g., sulfate and ammonium) and organic components (e.g., organic carbon) in PM_2.5_ are significantly associated with cardiovascular mortality [20]. Here, we hypothesized that exposure to primary PM (PPM) and SPM (sulfate, nitrate, ammonium, and SOA) could worsen atherothrombotic responses, such as the changes in indicators relevant to ceramide metabolism, inflammation response, and blood coagulation. We further explored which sources of organic aerosols (OA), such as primary OA (POA) and SOA, might be more responsible for the atherothrombotic changes. Finally, we examined the mediating roles of ceramide metabolism and inflammatory responses in PPM and SPM exposures associated with changes in blood coagulation.

## 2. Materials and Methods

### 2.1. Study Population

A prospective panel study was performed between September 2019 and January 2020 in Beijing, China. Young and healthy adults were investigated regularly through four clinical visits in this panel study. Participants who had any pre-existing chronic diseases were excluded. In total, 152 non-smoking healthy participants of 18 years or older were recruited (89% of participants living in student dormitories on campus). Upon each clinical visit, questionnaires were used to collect information about participants’ health status, medicine intake, smoking, and drinking status. Physical activity was assessed by the long International Physical Activity Questionnaire (IPAQ), and metabolic equivalent task (MET) score was calculated based on IPAQ guideline. Sleep quality was estimated by the Pittsburgh Sleep Quality Index (PSQI). Moreover, blood and urine samples were also collected during each clinical visit. The study protocol was approved by the Institutional Review Board of Peking University Health Science Center (PUHSC) in Beijing, China. All participants provided written informed consent.

### 2.2. Exposure Measurements

Ambient PM_2.5_ aerosol, including OA, sulfate (SO_4_^2−^), nitrate (NO_3_^−^), ammonium (NH_4_^+^), and chloride (Cl^−^), were measured by a time-of-flight aerosol chemical speciation monitor (ToF-ACSM, TOFWERK, Thun, Kanton Bern, Switzerland.) at the tower branch of Institute of Atmospheric Physics (IAP), Chinese Academy of Sciences (39°58′28″ N, 116°22′16″ E). Organics were further deconvolved into POA and SOA factors from various sources and processes using positive matrix factorization (PMF) [21,22,23]. The detailed operation and calibration of the ToF-ACSM instrument, as well as sources resolved by the PMF approach, are provided in the Supplementary Materials. Briefly, in the PMF solutions from 2 to 8 factors, the 4-factor solution so far has served as the most interpretable approach, which included POA (including cooking organic aerosol [COA] and fossil fuel-related OA [FFOA]) and SOA (including less oxidized oxygenated OA [LO-OOA] and more oxidized oxygenated OOA [MO-OOA]). Each factor was characterized by the typical prominent peaks indicative of different sources and properties [24]. In this study, chloride (Cl^−^) and POA were categorized as PPM, while secondary inorganic aerosols (SO_4_^2−^, NO_3_^−^, NH_4_^+^) and SOA were classified as SPM [24,25]. For quality assurance of ToF-ACSM, we repeated the signal-to-mass calibration using ammonium nitrate (NH_4_NO_3_) particles of known size and concentration at least every 8 weeks during normal operation and with increased frequency following a venting of the vacuum chamber [26].

### 2.3. Health Outcomes and Biomarker Measurements

Serum samples for each participant who fasted overnight were obtained from 8 a.m. to 12 a.m. during each clinical visit and stored at −80 °C. The targeted High-Performance Liquid Chromatography/Mass Spectrum (HPLC/MS) was used to measure concentrations of serum ceramides (Cer C16:0, Cer C18:0 and Cer C24:1) with a Kinetex^®^ C18 LC column (P/N 00D-4725-AN; Phenoemex, Torrance, CA, USA). Citrullinated histone H3 (H3Cit) was measured by a Citrullinated Histone H3 ELISA Kit (501620, Cayman, MI, USA). Circulating extracellular DNA levels were measured by a Quant-iT™ PicoGreen^®^ dsDNA Assay Kit (P7589, Invitrogen, Waltham, MA, USA). Myeloperoxidase (MPO) was analyzed by a Human Premixed Multi-Analyte Luminex Kit (RD-LXSAHM-17, R&D Systems, Minneapolis, MN, USA). The assays were measured on Luminex^®^ 200™ Instrument System (Luminex Corporation, Austin, TX, USA) with xPONENT software set according to the assay protocol. Serum levels of biomarkers indicative of systemic inflammation (regulated upon activation normal T cell expressed and secreted [RANTES], MCP-1, soluble CD163 [CD163]), and lipoprotein-associated phospholipase A2 [Lp-PLA2]) and coagulation (P-selectin and plasminogen activator inhibitor-1 [PAI-1]) were also analyzed using the Human Premixed Multi-Analyte Luminex Kits (RD-LXSAHM-17, R&D Systems, Minneapolis, USA). Serum levels of D-dimers were analyzed by a RayBio^®^ Human D-Dimer ELISA Kit (ELH-DDIMER, Ray Biotech, Norcross, GA, USA). Urinary cortisol concentrations were analyzed using an ELISA kit (LH-E10220HU, LIUHEBIO, Wuhan, China). Urinary cotinine, a metabolite of nicotine with a half-life of 16–20 h, was analyzed by a commercially available ELISA approach (DG11013H, Dogesce, Beijing, China) to assess recent exposure to environmental tobacco smoke. Both urinary cortisol and cotinine concentrations were adjusted by urinary creatinine (Cr) concentrations for further analyses and reported as ng/mg Cr. The quality control (QC) of biomarker measurements was also performed [27,28,29], and more details are provided in the Supplementary Materials. For instance, accuracy and precision for MCP-1 measurements were ensured with 101.1% sample recovery and replication of less than 20% variable coefficient (average 2.9%).

### 2.4. Statistical Analysis

#### 2.4.1. Demographic Characterization

The demographic characteristics of participants were described by the mean (standard deviation, SD) and median (interquartile range, IQR). Normal distribution was tested by Shapiro–Wilk test and all biomarkers (except dsDNA) were log-transformed. Differences in characteristics and measured biomarkers by gender were estimated by *t*-test. Spearman correlation coefficients were also performed for PM_2.5_ components, meteorological parameters, and measured biomarkers.

#### 2.4.2. Linear Mixed-Effect Models

The associations between ambient PPM and SPM and measured biomarkers were estimated by linear mixed-effect (LME) models, with various exposure metrics including pollutant concentrations in the moving averages of up to 72 h before the clinic visits (0 to 1 h [1 h average], 0 to 2 h [2 h average], 0 to 3 h [3 h average], 0 to 6 h [6 h average], 0 to 18 h [18 h average], 0 to 24 h [24 h average], 0 to 48 h [48 h average], and 0 to 72 h [72 h average]).

For all LME models, the fixed effects were introduced as health outcomes adjusted for all covariables, and potential correlation within each participant was accounted for by including a random intercept. Additionally, given the measures at unequal time intervals in this study, the continuous first-order autoregressive covariance structure was added into the models to control for the potential decay of correlation and time between repeated measures within each participant. Confounding variables for air pollution exposure and health outcomes were chosen by the directed acyclic graph (Figure S3). Finally, age, sex, month of the blood withdrawal, day of the week, hour of the blood withdrawal, physical activity, ambient temperature, and relative humidity were selected in the main LME models. The moving averages of temperature and relative humidity up to 7 days were both included in LME models and fitted with the natural spline function with less than 6 degrees of freedom according to Akaike’s Information Criterion. 

#### 2.4.3. Mediation and Sensitivity Analyses

To further assess the mediating roles of ceramides in associations between PPM and SPM and inflammation or coagulation, we performed mediation analyses with a single mediator based on LME models. We first normalized mediators (ceramide metabolic biomarkers) and the outcome measurements (inflammation and coagulation biomarkers). Two LME models were fitted with random intercepts in the single-mediator model, with one for associations of pollutants with mediators and associations of pollutants and another for outcome measures adjusted through designated mediators (the indirect effects). The confidence interval was calculated by the quasi-Bayesian Monte Carlo method with 1000 times of simulation based on normal approximation. This analysis estimated the total effect (the effect of pollutants on outcomes ignoring any mediators), the direct effects (the associations of pollutants with outcomes), the indirect effects, and the proportion of mediation (the proportion of the total effect due to a mediator). Moreover, we further performed the mediating role of inflammation (NETs and systemic inflammation) in associations of PM_2.5_ components and source-specific OA exposures with coagulation. All packages of lme4 and mediation were performed in the R software (version 4.1.1). Sensitivity analyses were performed by further adjusting for ambient PM_2.5_ concentration in the main LME models to examine the robustness of the observed associations. Given that data on PM_2.5_ mass concentrations were not available from ToF-ACSM, we therefore obtained hourly concentrations of ambient PM_2.5_ as well as meteorological parameters (ambient temperature and relative humidity) measured at the PUHSC School of Public Health Building (39°58′58″ N, 116°20′52″ E), which is within 1.5 km from the tower branch of the IAP (as shown in Figure S1). To assess coherence between different sites, a Pearson correlation test was conducted, and the results suggested that PM_2.5_ measured at the PUHSC campus and the sum of all components determined at IPA were highly correlated with a correlation coefficient of 0.96 (as shown in Figure S2).

The results are reported as the percentage changes of biomarkers and 95% confidence intervals (*CI*s) associated with IQR increases in concentrations of PM_2.5_ components (Table S1). Statistical significance was determined as *p*-value < 0.05, and a Bonferroni correction was performed at a significance level of *p*-value < 0.0026 (0.05/19), correcting for the number of measured biomarkers to control for potential type I error rate. All analysis was performed in R (version 4.1.1).

## 3. Results

**Figure 1:**
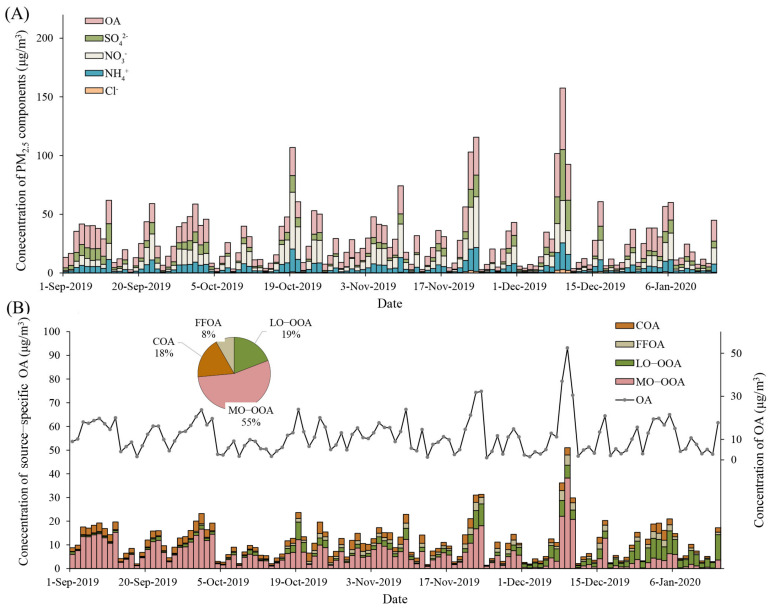
Time series of PM_2.5_ components (**A**) and source-specific OA (**B**). The gray line presented in (**B**) indicates the characteristics of the OA concentrations during the study period. The organic and inorganic components in PM_2.5_ were measured at the tower branch site of the Institute of Atmospheric Physics. Abbreviations: PM_2.5_, particulate matter in diameter < 2.5 μm; OA, organic aerosol; SO_4_^2−^, sulfate; NO_3_^−^, nitrate; NH_4_^+^, ammonium; Cl^−^, chloride; COA, cooking organic aerosol; FFOA, fossil fuel-related organic aerosol; LO-OOA, a less oxidized oxygenated organic aerosol; MO-OOA, a more oxidized oxygenates organic aerosol.

The baseline characteristics of participants and measured biomarkers are shown in Table 1. In this population of 152 non-smoking healthy participants (102 women and 50 men), the mean (SD) age and body mass index (BMI) were 23.9 (2.4) years old and 21.6 (2.7) kg/m^2^, respectively. Descriptive statistics of PM_2.5_ components and source-specific OA are displayed in Table S1. As shown in Figure 1, we found large day-to-day variations in concentrations of PM_2.5_ components and source-specific OA over the study period. For instance, daily average concentrations varied from 1.5 to 52.4 μg/m^3^ for OA and 0.2 to 38.3 μg/m^3^ for MO-OOA. It also showed that the day-to-day variations were overall similar for all SPM and source-specific OA over the study period. Using PMF analyses, we identified two sources of POA, including cooking and fossil fuel-related emissions and two sources of secondary OA, including atmosphere transportation and transformation. Among the identified sources, SOA (LO-OOA and MO-OOA) accounted for more than 60% of the measured OA (as shown in Figure 1B). The Spearman’s correlation coefficients among PM_2.5_ components and source-specific OA and measured biomarkers are shown in Figures S4 and S5, respectively. Most components were positively correlated, despite the fact that FFOA negatively correlated with SOA.

**Figure 2:**
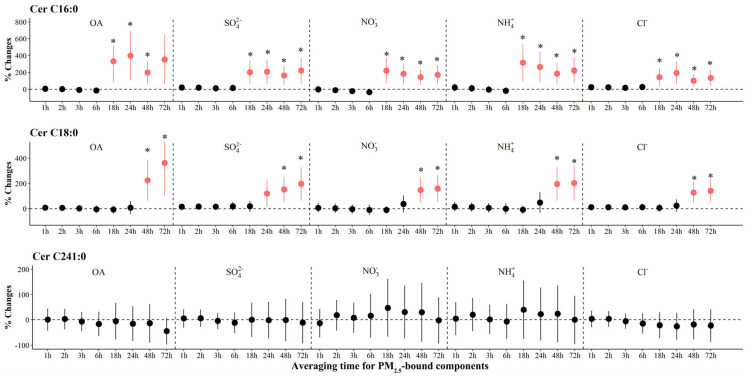
The adjusted percentage changes in ceramides associated with per IQR increment in PM_2.5_ components. Averaging time represents the mean of PM_2.5_ component concentrations over the 1 to 72 h (3 days) prior to each participant’s clinic visit. The IQR concentrations for averaged concentration on the full hour of blood draws of OA, SO_4_^2−^, NO_3_^−^, NH_4_^+^, and Cl^−^ are 10.4 μg/m^3^, 5.0 μg/m^3^, 9.0 μg/m^3^, 5.0 μg/m^3^, and 0.2 μg/m^3^, respectively. Error bars indicated 95% CI. Significant association is shown in red (*p* < 0.05). Bonferroni corrections with significance (*p* < 0.0026) are indicated by asterisks. Abbreviations: IQR; interquartile range; PM_2.5_, particulate matter in diameter < 2.5 μm; Cer, ceramides; OA, organic aerosol; SO_4_^2−^, sulfate; NO_3_^−^, nitrate; NH_4_^+^, ammonium; Cl^−^, chloride.

**Figure 3:**
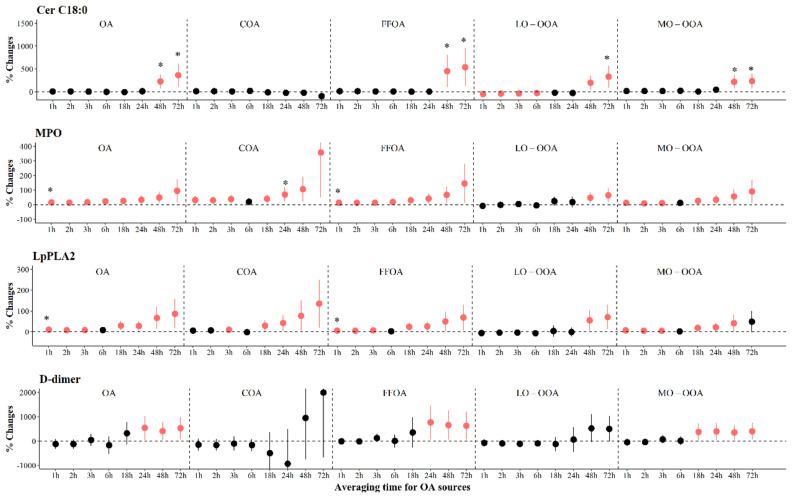
The adjusted percentage changes in selected biomarkers associated with per IQR increment in moving average concentrations of source-specific OA. Averaging time represents the mean of source-specific OA concentrations over the 1 to 72 h (3 days) prior to each participant’s clinic visit. The IQR concentrations for averaged concentration on the full hour of blood draws of OA, COA, FFOA, LO-OOA, and MO-OOA are 10.4 μg/m^3^, 1.8 μg/m^3^, 1.1 μg/m^3^, 2.4 μg/m^3^, and 6.3 μg/m^3^, respectively. Error bars indicate 95% CI. Significant association is shown in red (*p* < 0.05). Bonferroni corrections with significance (*p* < 0.0026) are indicated by asterisks. Abbreviations: IQR; interquartile range; PM_2.5_, particulate matter with an aerodynamic diameter smaller than 2.5 μm; Cer, ceramides; MPO, myeloperoxidase; Lp-PLA2, lipoprotein-associated phospholipase A2; OA, organic aerosol; COA, cooking organic aerosol; FFOA, fossil fuel-related organic aerosol; LO-OOA, a less oxidized oxygenated organic aerosol; MO-OOA, a more oxidized oxygenates organic aerosol.

The associations of PM_2.5_ components and source-specific OA with ceramides are shown in Figure 2 and Figure S6. We observed significant increases in levels of Cer C16:0 and Cer C18:0 associated with IQR increases in all PM_2.5_ components at various periods. The largest increase [397.9% (95% CI: 105.0, 690.8)] in Cer C16:0 was observed for OA exposure at 24 h prior to the clinic visit, and the largest increase [362.5% (95% CI: 105.6, 619.3)] in Cer C18:0 was observed for OA exposure at 72 h prior to clinic visits. We observed significant increases of 199.0% to 541.0% in Cer C18:0 associated with IQR increases in most sources of OA at 48 to 72 h prior to the clinic visit (Figure 3). However, associations of PM_2.5_ components and source-specific OA were not statistically significant for Cer C24:1.

**Figure 4:**
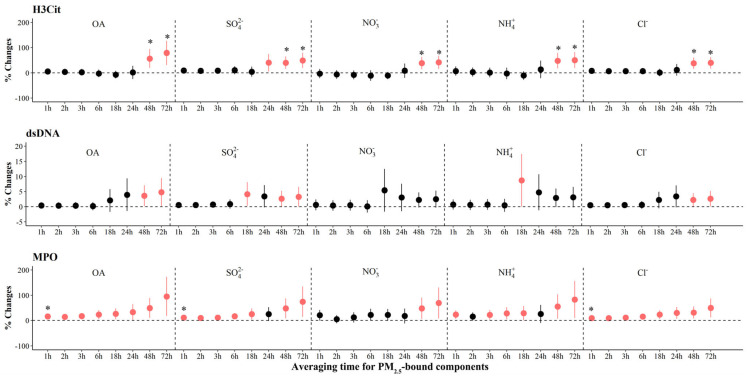
The adjusted percentage changes in NETs associated with per IQR increment in PM_2.5_ components. Averaging time represents the mean of PM_2.5_ components concentration over the 1 to 72 h (3 days) prior to each participant’s clinic visit. The IQR concentrations for averaged concentration on the full hour of blood draws of OA, SO_4_^2−^, NO_3_^−^, NH_4_^+^, and Cl^−^ are 10.4 μg/m^3^, 5.0 μg/m^3^, 9.0 μg/m^3^, 5.0 μg/m^3^, and 0.2 μg/m^3^, respectively. Error bars indicate 95% CI. Significant association is shown in red (*p* < 0.05). Bonferroni corrections with significance (*p* < 0.0026) are indicated by asterisks. Significant association is shown in red (*p* < 0.05). Abbreviations: IQR; interquartile range; PM_2.5_, particulate matter in diameter < 2.5 μm; NET, neutrophil extracellular traps; H3Cit, citrullinated histone H3; dsDNA, double-stranded DNA; MPO, myeloperoxidase; OA, organic aerosol; SO_4_^2−^, sulfate; NO_3_^−^, nitrate; NH_4_^+^, ammonium; Cl^−^, chloride.

**Figure 5:**
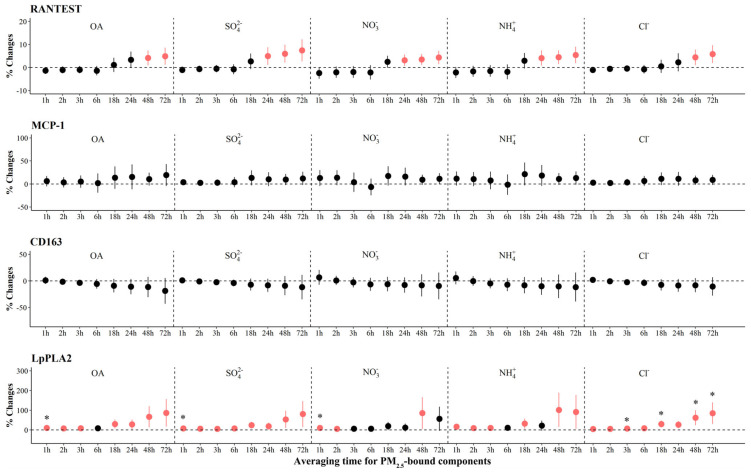
The adjusted percentage changes in systemic inflammation biomarkers with per IQR increment in PM_2.5_ components. Averaging time represents the mean of PM_2.5_ component concentrations over the 1 to 72 h (3 days) prior to each participant’s clinic visit. The IQR concentrations for averaged concentration on the full hour of blood draws of OA, SO_4_^2−^, NO_3_^−^, NH_4_^+^, and Cl^−^ are 10.4 μg/m^3^, 5.0 μg/m^3^, 9.0 μg/m^3^, 5.0 μg/m^3^, and 0.2 μg/m^3^, respectively. Error bars indicate 95% *CI*. Significant association is shown in red (*p* < 0.05). Bonferroni corrections with significance (*p* < 0.0026) are indicated by asterisks. Abbreviations: IQR; interquartile range; PM_2.5_, particulate matter in diameter < 2.5 μm; RANTES, regulated upon activation normal T cell expressed and secreted; MCP-1, monocyte chemoattractant protein-1; Lp-PLA2, lipoprotein-associated phospholipase A2; OA, organic aerosol; SO_4_^2−^, sulfate; NO_3_^−^, nitrate; NH_4_^+^, ammonium; Cl^−^, chloride.

As shown in Figure 4, we observed significant increases of 38.2% (95% CI: 15.7, 60.7) to 78.8% (95% CI: 30.7, 127.0) in H3Cit associated with IQR increases in most PM_2.5_ components at various periods, with the largest effects associated with OA, SO_4_^2−^, NO_3_^−^, NH_4_^+^, and Cl^−^ in the 72 h before the clinic visit. We also observed significant increases in MPO associated with IQR increases in all components, ranging from 3.1% (95% CI: 0.7, 5.6) to 7.5% (95% CI: 2.7, 12.3) at 24 to 72 h prior to clinic visits. In contrast, we observed significant decreases of −6.7% to −2.5% in MPO with per IQR increase in LO-OOA at 1 to 3 h (Figure 3 and Figure S7). For systemic inflammation indicators, we observed significant increases of 2.6% (95% CI: 1.0, 4.2) to 7.6% (95% CI: 2.6, 12.6) in RANTES associated with IQR increases in most PM_2.5_ components at various periods, with the largest effects associated with OA, SO_4_^2−^, NO_3_^−^, NH_4_^+^, and Cl^−^ in the 72 h before the clinic visit (Figure 5). For LpPLA2, we observed significant increases of 5.7% (95% CI: 1.7, 9.7) to 81.1% (95% CI: 15.5, 146.6) associated with IQR increases in SO_4_^2−^ at 1 to 72 h before clinic visits, and 29.8% (95% CI: 3.2, 56.4) to 135.3% (95% CI: 20.2, 250.4) with IQR increases in COA at 18 to 72 h before clinic visits (Figure 3 and Figure S8). However, associations of PM_2.5_ components and source-specific OA were not statistically significant for MCP-1 and CD163.

**Figure 6:**
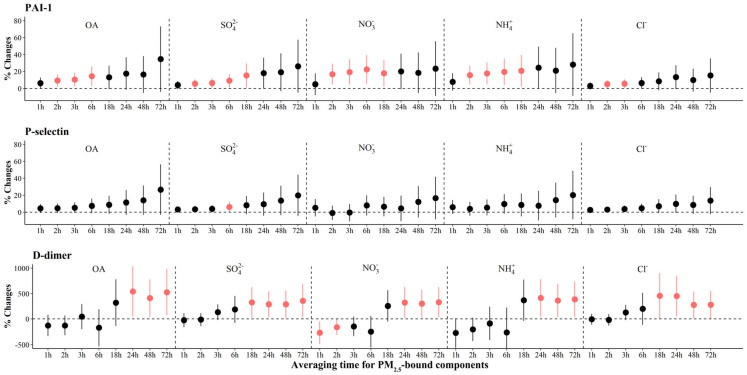
The adjusted percentage changes in coagulation biomarkers per IQR increment in PM_2.5_ components. Averaging time represents the mean of PM_2.5_ component concentrations over the 1 to 72 h (3 days) prior to each participant’s clinic visit. The IQR concentrations for averaged concentration on the full hour of blood draws of OA, SO_4_^2−^, NO_3_^−^, NH_4_^+^, and Cl^−^ are 10.4 μg/m^3^, 5.0 μg/m^3^, 9.0 μg/m^3^, 5.0 μg/m^3^, and 0.2 μg/m^3^, respectively. Error bars indicate 95% CI. Significant association is shown in red (*p* < 0.05). Abbreviations: IQR; interquartile range; PM_2.5_, particulate matter in diameter < 2.5 μm; PAI-1, plasminogen activator inhibitor-1; OA, organic aerosol; SO_4_^2−^, sulfate; NO_3_^−^, nitrate; NH_4_^+^, ammonium; Cl^−^, chloride.

We observed significant increases of 5.1% (95% CI: 0.7, 9.4) to 22.5% (95% CI: 5.7, 39.2) in PAI-1 associated with IQR increases in most PM_2.5_ components, with the largest effects associated with NO_3_^−^ in the previous 6 h before the clinic visits (Figure 6). For source-specific OA, we observed significant increases of 7.3% (95% CI: 0.9, 13.7) to 119.4% (95% CI: 3.0, 160.0) in PAI-1 associated with IQR increases in all sources but LO-OOA (Figure 3 and Figure S9). The largest increase [541.5% (95% CI: 47.4, 1035.7)] in D-dimers was observed for OA exposure at 24 h prior to the clinic visits, and significant increases were observed for FFOA and MO-OOA exposure. However, significant increases in P-selectin were only observed for SO_4_^2−^ 6 h prior to the clinic visits.

Sensitivity analyses for associations between measured biomarkers and PM_2.5_ components are shown in Figures S10–S13. After further adjusting for PM_2.5_ concentrations, we observed significant increases in some biomarkers (e.g., Cer 18:0, H3Cit) associated with IQR increases in most PM_2.5_ components at 1 to 6 h prior to clinic visits. Compared with the main models, no significant increases in Lp-LPA1 and PAI-1 were observed, with IQR increases in PM_2.5_ components. Moreover, we also observed that significant decreases in biomarkers relevant to inflammation (e.g., RANTES) were associated with IQR increases in COA 1 to 3 h before clinic visits.

**Figure 7:**
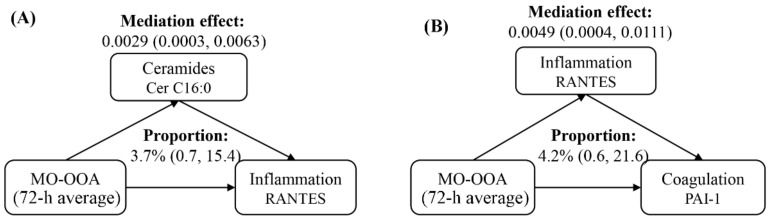
Mediation analyses of ceramides on inflammation biomarkers (**A**) and inflammation biomarkers on coagulation biomarkers (**B**) following PM_2.5_ components and source-specific OA exposure. Averaging time represents the mean of source-specific concentrations over the 1 to 72 h (3 days) prior to each participant’s clinic visit. Abbreviations: PM_2.5_, particulate matter in diameter < 2.5 μm; MO-OOA, a more oxidized oxygenates organic aerosol; RANTES, regulated upon activation normal T cell expressed and secreted; PAI-1, plasminogen activator inhibitor.

Mediation analyses indicated that ceramides could mediate the effects of PM_2.5_ components exposure on various metrics of inflammatory response (Figure 7A). In specific, we estimated that Cer C16:0 could mediate 3.7% of the association between 72 h averages of MO-OOA and RANTES (Figure 7A) and medicate 5.3% of the association between 72 h averages of NH_4_^+^ and RANTES (Table S2). Moreover, RANTES, indicative of systemic inflammation, could mediate up to 8.3% of PAI-1 changes with PM_2.5_ components and source-specific OA (Figure 7B). The summary results of mediation analyses are presented in Table S2.

## 4. Discussion

In this study, we observed the effects of exposure to SPM (SO_4_^2−^, NO_3_^−^, NH_4_^+^, and SOA) and PPM (Cl^−^ and POA) in PM_2.5_ on dysfunctional ceramide metabolism (Cer C16:0 and Cer C18:0), increased formation of NETs (increases in H3Cit, dsDNA and MPO), potentiated systemic inflammation (increases in RANTES, and Lp-PLA2), and altered blood coagulation (increases in PAI-1, P-selectin, and D-dimer). OA from fossil fuel and secondary transformation was found to have a significant impact on the worsened pathophysiology of atherothrombosis following short-term exposure in prior hours to days. In addition, exposure to PM_2.5_ components and source-specific OA could contribute to inflammatory responses mediated by ceramide metabolisms and coagulation dysfunction mediated by inflammatory responses. Our findings provide evidence that PM from anthropogenic emissions could be more responsible for cardiovascular dysfunction, which could highlight the importance of reducing air pollution emissions from combustion sources.

### 4.1. The Association between Ionic Aerosols and Biomarkers Relevant to Pathophysiology of Atherothrombosis

Our collective findings indicated that higher levels of ionic aerosols (SO_4_^2−^, NO_3_^−^, NH_4_^+^ in SPM and Cl^−^ in PPM) were significantly associated with atherothrombotic responses. It was reported that water-soluble ions (e.g., SO_4_^2−^, NO_3_^−^, and NH_4_^+^) could account for 30–60% mass fractions of PM_2.5_ in Beijing, mainly produced by anthropogenic sources [30,31,32]. Moreover, SO_4_^2−^, NO_3_^−^, NH_4_^+^, and Cl^−^ were described as the first principal components (>0.8 factor-loading) in principal component analysis for main sources of PM_2.5_ in Beijing, which support the primary influence of secondary aerosols from the anthropogenic sources (e.g., coal combustion) [33]. Recent studies have shown that SO_4_^2−^ exposure could increase the risks of hospitalizations and morbidity of cardiovascular diseases [34,35]. Abundant evidence in humans has suggested that short-term exposure to PM_2.5_ could be associated with increases in circulating biomarkers related to ceramides, inflammation, and coagulation [36,37]. We observed significantly positive associations between PM_2.5_ components and most biomarkers 18 h prior to the clinic visits. Previous studies reported that the acute effects of PM_2.5_ on ceramides were observed after a 1-day moving average of exposure [37,38]. For biomarkers indicative of NETs, we observed significant positive associations of PM_2.5_ components with MPO after a 1 h moving average, compared to significant effects appearing in H3Cit and dsDNA after an 18 h moving average of exposure. One possible explanation is that MPO is one of the major neutrophil bactericidal proteins and can be released upon neutrophil activation, while MPO is also an enzyme involved in the formation of NETs [33]. In addition, MPO may drive chromatin decondensation and the release dsDNA and H3Cit [39,40]. For coagulation, we observed the positive effects of PM_2.5_ components on PAI-1 occurred within 2 to 18 h prior to clinic visits. Largely consistent with our findings, a previous study showed that the effects of PM_2.5_ on PAI-1 occurred within 0 to 6 h and disappeared after 24 h [41]. In this study, we also found a decrease in D-dimer with IQR increases in NO_3_^−^ at 1 to 3 h before the clinic visits. PAI-1 could inhibit the activity of plasminogen, which is the main enzyme in the process of fibrinolysis, leading to an increase in fibrinolysis [42]. At the same time, D-dimers are specific crosslinked fibrin derivatives that are the product of plasmin-mediated fibrinolytic degradation [39,40], which could explain the decreases in D-dimers at early exposure periods. Some studies found that exposure to inorganic components of PM_2.5_ is associated with elevated levels of biomarkers related to inflammation. For example, a panel study among 43 healthy students showed that short-term exposure to PM_2.5_ components, including Cl^−^, was significantly associated with increased levels of IL-8 [41]. Mechanistically, recent evidence of NETs is present in thrombi retrieved from thrombosis patients [43]. NETs could promote platelet adhesion, activation and aggregation, and active plaque remodeling, potentially triggering atherothrombosis [44]. As a key regulator of fibrinolysis, elevated expression of PAI-1 can promote the formation of plaques and favor plaque rupture [45]. An in vitro cell culture study indicated that pharmacological inhibition of PAI-1 may block PM_2.5_-induced PAI-1 and pro-fibrogenic signaling, thereby inhibiting the effects of PM_2.5_ on atherosclerosis [46]. Taken together, our study extends our understanding of potential pathophysiological pathways of cardiovascular events posed by PPM and SPM, which suggest that recent exposure to ionic aerosols may prompt atherothrombotic responses. 

### 4.2. The Association between OA and Biomarkers Relevant to Pathophysiology of Atherothrombosis

In this study, we observed that significant increases in indicators of ceramide metabolism, coagulation, and inflammation were associated with higher levels of OA exposure. Epidemiologic studies have shown that exposure to PM_2.5_ organics was significantly associated with increased mortality of cardiovascular diseases [47]. A multicenter study in China has also found positive associations between sub-clinical outcomes of cardiovascular diseases and exposure to organic matter in PM [48]. A panel study among 60 elderly subjects with coronary artery disease showed that organic carbon but not secondary organic carbon exposure was significantly associated with an increase in soluble P-selectin [49]. Our previous study also indicated that PM_2.5_ organic components (polycyclic aromatic hydrocarbons) could promote inflammatory response (monocyte/macrophage activation), which is in line with the findings of this study [36]. Mechanistically, previous studies indicated that organic chemicals in PM may lead to the production of reactive oxygen species in various cells of the lungs, blood, and vascular tissues, thereby promoting inflammatory response [50]. 

### 4.3. The Association between Sources of OA and Biomarkers Relevant to Pathophysiology of Atherothrombosis

It is important to understand which specific sources of PM_2.5_ could possess adverse health effects from the scientific standpoints and regulatory perspectives. The present study found that OA from combustion emissions, including fossil fuel combustion, were significantly associated with atherothrombotic responses. Additionally, we also found significant associations of changes in biomarkers with SOA (LO-OOA and MO-OOA), which indicated that secondary transformation and transportation related to PM could possess adverse impacts on cardiovascular health. The sources of atmospheric PM and its precursors over the winter in Beijing were mainly dominated by combustion emissions (e.g., industrial coal combustion burning and traffic) [24,51]. SOA can be formed by the photochemical reaction of volatile organic compounds and further condensation of the oxidation products onto the particles, mainly sourced from anthropogenic emissions [52]. Importantly, secondary formation could contribute more to PM_2.5_ concentrations during polluted periods compared with primary emissions [51]. Previous animal studies indicated that combustion-derived particulate matter is capable of triggering atherogenesis progressions [53,54,55]. For combustion sources, an in vivo study found that 7-day diesel exhaust exposure could induce the production of ceramides in the mouse lung [11]. Other evidence indicates that a mixture of diesel engine and gasoline engine vehicle emissions exposure could induce inflammations characterized by increases in MCP-1 levels [56]. For SOA, a cross-sectional study found a stronger association between per unit mass of SOA exposure and county-level cardiorespiratory death risks than that of PM_2.5_ exposure [7]. A panel study also showed that SOA was significantly associated with increases in the level of IL-6 (indicative of inflammatory response) [57]. Acellular assays of laboratory-generated SOA from terpenes, isoprene, and aromatics have shown that the oxidative potential of SOA could approach that of ambient PM_2.5_ and known hazardous air pollutants such as diesel exhaust particles, resulting in oxidant stress [58]. However, we found decreases in some inflammatory indicators (e.g., H3Cit, RANTES) associated with high levels of LO-OOA exposure at prior 1 to 6 h before clinic visits. One possible explanation is that chemical compounds (e.g., oxygenated aromatic anhydride moieties) in LO-OOA may exert immunosuppressive effects [57]. Another possible explanation is that LO-OOA is lower oxygenated than MO-OOA, which suggests that there is more time to change biomarkers. In this study, we observed that dysregulated ceramide metabolism could mediate the associations of increased systemic inflammation responses with MO-OOA exposure. We also observed that inflammatory responses could also mediate coagulation dysregulation, which is attributed to MO-OOA. Previous studies described ceramides as potent pro-inflammatory agents which could induce the activation of pro-inflammatory transcription factor NF-κB [59]. NF-κB can regulate the expression of more than 150 different genes in mammalian cells, leading to the upregulation of many other genes involved in inflammatory responses, including chemokines such as RANTES [60]. Herein, our collective findings suggest that OA-associated increases in inflammatory responses may be mediated through dysregulated ceramide pathways. Taken together, our results indicated that short-term exposure to SPM from combustion emissions could play an important role in the pathophysiology of atherothrombosis, including ceramide metabolisms, inflammation, and blood coagulation.

### 4.4. Strengths and Limitations

Our study has several advantages. First, the repeated measurement study design allowed each participant to serve as their own control to better control for potential confounding factors across individuals. To account properly for potential confounders, we also hypothesized a network of causal relationships in the DAG. Second, PM_2.5_ component concentrations were measured by real-time ToF-ACSM with high temporal resolution [61]. Third, we applied mediation analyses to elucidate the possible pathophysiological mechanisms of PPM-associated and SPM-associated atherothrombotic responses. Fourth, we observed significant changes in biomarker levels associated with PPM and SPM exposure in a homogenous group of nonsmoking healthy and young adults by eliminating potential confounding effects from medication use, pre-existing disease, and age-related susceptibility.

However, there are also several study limitations worth noting. The measurement of PM_2.5_ component exposure relying on one fixed monitoring station might result in non-different exposure misclassification, leading to associations toward null. In this study, we focused on assessing the associations with sources of OA, and the effects attributed to sources of other PM components are needed to evaluate in future investigations. Moreover, the concentrations of the PM_2.5_ components and PM_2.5_ mass were measured at different locations, which may introduce potential bias in the interpretation of the estimates between PM_2.5_ and source-specific components. However, the two monitoring sites are located in adjacent neighborhoods within 2 km, and measurements were observed with high correlations. Further, most participants in this study were young and healthy, which may limit the generalizability of our study findings to other individuals (e.g., the elderly).

## 5. Conclusions

In this study, we have shown that short-term exposure to PPM and SPM from combustion was significantly associated with potentiating pro-inflammatory action (including promoting NETs formation and heightening systemic inflammation) and dysregulating coagulation, which could be mediated, in part, through ceramide metabolism. Our findings provide insights into biological mechanisms linking ambient PM to the worsening of atherothrombotic responses and highlight the significance of controlling anthropogenic PM emissions to reduce the burden of cardiovascular diseases. 

## Figures and Tables

**Table 1:** Descriptive statistics of the participant characteristics and measured biomarkers.

Variables	Total (N = 152)		Women (N = 102)		Men (N = 50)	
	Mean (SD)	Median (IQR)	Mean (SD)	Median (IQR)	Mean (SD)	Median (IQR)
Characteristics						
Age, years	23.9 (2.4)	23.8 (2.2)	23.6 (2.2)	23.8 (2.8)	24.4 (2.7)	24.1 (2.3)
BMI, kg/m^2^	21.6 (2.7)	21.4 (3.4)	20.9 (2.6)	20.6 (23.2)	23.0 (2.4)	22.6 (2.8)
WHR	0.8 (0.1)	0.8 (0.10)	0.8 (0.05)	0.76 (0.07)	0.8 (0.04)	0.8 (0.05)
MET, minutes/week	1884 (1794)	1386 (1962)	1763 (1700)	1224 (1889)	2126 (1951)	1551 (2330)
PSQI	4.9 (2.3)	5.0 (3.0)	4.7 (2.4)	5.0 (3.0)	5.3 (2.9)	5.0 (4.0)
Cortisol, ng/mg creatinine	1.8 (1.2)	1.5 (1.0)	2.0 (1.3)	1.7 (1.2)	1.6 (0.8)	1.3 (0.6)
Cotinine, ng/mg creatinine	1.8 (1.2)	1.5 (1.1)	2.0 (1.3)	1.6 (1.3)	1.5 (0.8)	1.2 (0.7)
Measured biomarkers						
Ceramides, μM						
Cer C16:0	22.9 (48.3)	8.8 (22.0)	25.5 (37.2)	5.9 (24.3)	14.4 (20.5)	7.4 (17.3)
Cer C18:0	27.9 (58.0)	10.3(28.6)	29.6 (30.2)	19.9 (40.6)	21.5 (30.3)	7.3 (21.5)
Cer C24:1	30.1 (49.5)	14.6 (31.6)	29.1 (50.6)	14.5 (28.2)	32.0 (47.5)	15.7 (38.7)
NETs, pg/mL						
H3Cit	4.8 (6.5)	3.2 (4.7)	4.5 (4.6)	3.3 (4.5)	5.5 (9.1)	3.0 (5.3)
dsDNA	163.4 (21.6)	162.4 (28.5)	157.0 (22.8)	154.4 (24.0)	175.5 (20.9)	173.9 (29.1)
MPO	18.7 (9.2)	17.0 (10.7)	17.8 (8.1)	16.6 (14.9)	23.0 (12.8)	19.4 (15.0)
Systemic inflammation, pg/mL						
RANTES	10.2 (3.8)	9.7 (4.3)	10.1 (3.8)	9.7 (4.0)	10.4 (3.7)	9.7 (5.2)
MCP-1	146.7 (67.2)	138.1 (46.2)	144.2 (78.1)	133.2 (43.6)	151.5 (36.7)	145.8 (46.3)
CD163	215.1 (87.1)	203.7 (129.0)	213.9 (82.6)	207.1 (121.1)	217.4 (95.6)	193.7 (146.5)
LpPLA2	40.3 (11.8)	39.6 (16.7)	38.5 (11.0)	36.7 (13.8)	44.0 (12.5)	44.7 (19.1)
Coagulation, pg/mL						
PAI-1	38.5 (140.9)	26.4 (17.6)	28.3 (15.1)	23.8 (15.5)	58.9 (242.0)	32.0 (21.6)
P-selectin	23.0 (5.4)	22.7 (7.7)	20.2 (3.7)	20.0 (5.0)	24.6 (5.2)	23.5 (8.5)
D-dimer, ng/mL	17.6 (45.3)	5.7 (13.7)	19.7 (53.7)	5.2 (15.6)	13.4 (19.3)	6.2 (11.1)

Descriptive statistics of demographic variables (except for age) were computed on the basis of all follow-ups. Mean (SD) and Median (IQR) are computed based on all repeated clinical visits of measured biomarkers. Abbreviations: BMI, body mass index; WHR, waist-to-hip ratio; MET, metabolic equivalent task; PSQI, Pittsburgh sleep quality index; Cer, ceramides; H3Cit, citrullinated histone H3; dsDNA, double-stranded DNA; MPO, myeloperoxidase; RANTES, regulated upon activation normal T cell expressed and secreted; MCP-1, monocyte chemoattractant protein-1; Lp-PLA2, lipoprotein-associated phospholipase A2; PAI-1, plasminogen activator inhibitor-1.

## Data Availability

Data is unavailable due to privacy or ethical restrictions.

## Supplementary Materials

The following supporting information can be downloaded at https://www.mdpi.com/article/10.3390/toxics12030225/s1, Online Methods; Table S1. Concentrations of ambient PM_2.5_ and its components during the study period.; Table S2. Results from single-mediator models; Figure S1. Maps of the sampling site and residential location of participants; Figure S2. The scatter plot of NR-PM_2.5_ to PM_2.5_ in each year from September 2019 to January 2020; Figure S3. Confounding variables for characterizing the associations between air pollution exposure and health outcomes selected by the directed acyclic graph; Figure S4. Spearman’s correlations between PM_2.5_ components and meteorological parameters; Figure S5. Spearman’s correlations of measured biomarkers; Figure S6. The adjusted percentage changes in ceramides associated with per IQR increment in source-specific OA; Figure S7. The adjusted percentage changes in NETs associated per IQR increment in source-specific OA; Figure S8. The adjusted percentage changes in pro-inflammation biomarkers associated with IQR increment in source-specific OA; Figure S9. The adjusted percentage changes in coagulation associated with IQR increment in source-specific OA; Figure S10. Percentage changes in ceramides associated with PM_2.5_ components (A) and source-specific OA (B) adjusted by PM_2.5_; Figure S11. Percentage changes in NETs associated with PM_2.5_ components (A) and source-specific OA (B) adjusted by PM_2.5_; Figure S12. Percentage changes in systemic inflammation associated with PM_2.5_ components (A) and source-specific OA (B) adjusted by PM_2.5_. Figure S13. Percentage changes in coagulation associated with PM2.5 components (A) and source-specific OA (B) adjusted by PM_2.5_.
