# Supplementary material for: Short-Term Effects of Primary and Secondary Particulate Matter on Ceramide Metabolism, Pro-Inflammatory Response, and Blood Coagulation
Source: Toxics. 2024 Mar 19;12(3):225. doi: 10.3390/toxics12030225 (PMC10974030; doi:10.3390/toxics12030225)
Supplement: Supplementary file 1 [file toxics-12-00225-s001.zip › toxics-2888075-supplementary.pdf]

Supplementary Materials

Short-Term Effects of Primary and Secondary Particulate Matter on Ceramide Metabolism, Pro-Inflammatory Response, and Blood Coagulation

Bin Zhang[1,2]†, Hongbing Xu[1,2]†, Xinghou He[1,2], Tong Wang[1,2], Mengyao Li[1,2], Xuyang Shan[1,2], Yutong Zhu[1,2], Changjie Liu[2,3], Qian Zhao[1,2], Xiaoming Song[1,2], Yele Sun[4], Lemin Zheng[2,3*], Wei Huang[1,3*]

[1]Department of Occupational and Environmental Health, Peking University School of Public Health, and Peking University Institute of Environmental Medicine, Beijing, 100191, China

[2]State Key Laboratory of Vascular Homeostasis and Remodeling, Peking University, Beijing, 100191, China

[3]Institute of Cardiovascular Sciences and Institute of Systems Biomedicine, Peking University School of Basic Medical Sciences, Beijing, 100191, China

[4]State Key Laboratory of Atmospheric Boundary Layer Physics and Atmospheric Chemistry, Institute of Atmospheric Physics, Chinese Academy of Sciences, Beijing, 100191, China

† Contributed equally to this work.

[*]Corresponding authors: Prof. Wei Huang, Department of Occupational and Environmental Health, Peking University School of Public Health, No. 38 Xueyuan Rd, Haidian District, Beijing, China, 100191. E-mail: whuang@bjmu.edu.cn. Prof. Lemin Zheng, Institute of Cardiovascular Sciences and Institute of Systems Biomedicine, Peking University School of Basic Medical Sciences, No. 38 Xueyuan Rd, Haidian District, Beijing, China, 100191. E-mail: zhengl@bjmu.edu.cn.

Contents

Online Methods..3

Table S1. Concentrations of ambient $PM_{2.5}$ and its components during the study period. 8

Figure S1. Maps of the sampling sites and residential location of participants. ©Google Earth....10

Figure S2. The scatter plot of NR-$PM_{2.5}$ to $PM_{2.5}$ in each year from September, 2019 to January, 2020. 11

Figure S3. Confounding variables for characterizing the associations between air pollution exposure and health outcomes selected by the directed acyclic graph..12

Figure S4. Spearman's correlations between $PM_{2.5}$ components and meteorological parameters..13

Figure S5. Spearman's correlations of measured biomarkers. S..14

Figure S6. The adjusted percentage changes in ceramides associated with per IQR increment in source-specific OA..15

Figure S7. The adjusted percentage changes in NETs associated with per IQR increment in source-specific OA..16

Figurer S8. The adjusted percentage changes in pro-inflammation biomarkers associated with IQR increment in source-specific OA...17

Figure S9. The adjusted percentage changes in coagulation associated with IQR increment in source-specific OA..18

Figure S10. Percentage changes in ceramides associated with $PM_{2.5}$ components (A) and source-specific OA (B) adjusted by $PM_{2.5}$..19

Figure S11. Percentage changes in NETs associated with $PM_{2.5}$ components (A) and source-specific OA (B) adjusted by $PM_{2.5}$..20

Figure S12. Percentage changes in systemic inflammation associated with $PM_{2.5}$ components (A) and source-specific OA (B) adjusted by $PM_{2.5}$. 21

Figure S13. Percentage changes in coagulation associated with $PM_{2.5}$ components (A) and source-specific OA (B) adjusted by $PM_{2.5}$..23

Online Methods

PMF for OA source apportionment

The PMF with PMF2.exe algorithm (v 4.2) [21] was performed on ACSM OA mass spectra following the procedures described in the previous study [22]. The PMF solutions from 2 to 8 factors were evaluated by checking the mass spectral profiles, diurnal variations, and temporal variations. The 4-factor solution is the most interpretable, including a cooking OA (COA), fossil fuel combustion OA (FFOA), less oxidized oxygenated OA (LO-OOA), and more oxidized OOA (MO-OOA).

The COA spectrum showed prominent ion peaks of *m/z* 55 and *m/z* 57, two marker *m/z*'s for COA. Consistently, the time series of COA correlated well with that of *m/z* 55 in both summer and winter. The two pronounced mealtime peaks in the diurnal cycle further supported that this factor is dominantly from cooking emissions. The FFOA mass spectrum is characterized by prominent unsaturated hydrocarbons, and PAH-related ion peaks, representing the sources of coal combustion and traffic exhaust. Consistently, FFOA correlated well with chloride, a tracer species for coal combustion in winter, and the diurnal patterns showed higher concentrations at nighttime than daytime. However, the two sources were not able to be separated due to the limited mass resolution of ACSM, and similar OA spectra between coal combustion and traffic OA [23].

Two different types of secondary OA were identified both of which showed high *m/z* 44 (dominantly CO_2^+), a tracer *m/z* for SOA. However, the temporal variations and diurnal cycles differed from each other greatly. While LO-OOA was highly correlated with nitrate, MO-OOA had a better correlation with sulfate, suggesting that LO-OOA is more representative of photochemical SOA, and MO-OOA is more from aqueous-phase production.

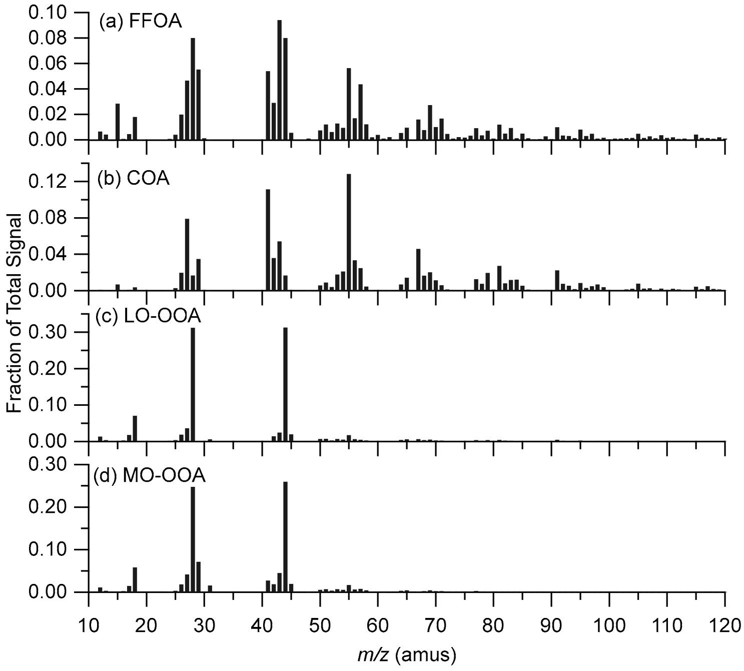

Figure. Mass spectra of four OA factors from PMF analysis.

Ceramide measurements

Serum samples were taken between 8 and 12 A.M. after an overnight fast and then stored at -80°C until analysis. We used the targeted HPLC/MS method to measure the concentrations of 5 ceramides including ceramide C16:0, C18:0, and C24:1. The analysis was performed using a Kinetex® C18 LC column (P/N 00D-4725-AN; Phenomenex, Torrance, CA). Mobile phase A was prepared with 5 mM ammonium acetate in 60% methamphetamine and 40% deionized water with 0.2% formic acid. Mobile phase B was prepared with 5 mM ammonium acetate in 60% methamphetamine and 40% isopropanol with 0.2% formic acid.

The quality control (QC) of biomarker measurements.

1) Ceramides

The targeted High Performance Liquid Chromatography/Mass Spectrum (HPLC/MS) with a Kinetex® C18 LC column (P/N 00D-4725-AN; Phenoemex, Torance, CA) was used to measure the serum concentration of ceramides (Cer C16:0, Cer C18:0 and Cer C24:1).

In this study, quality control of two ceramides were as follows:

a. Precision of the instrument

Cer C16:0	6.32
Cer C24:1	7.96

b. Recovery rates of the method

Biomarker	**Recovery rates (%)**
Cer C16:0	117.84
Cer C24:1	106.05

c. Stability of the method

Biomarkers	Intra-day CV（%）	Intra-day CV（%）	Intra-day CV（%）
Cer C16:0	7.55	9.06	5.73
Cer C24:1	8.76	7.34	8.21

d. Principal component analysis, PCA

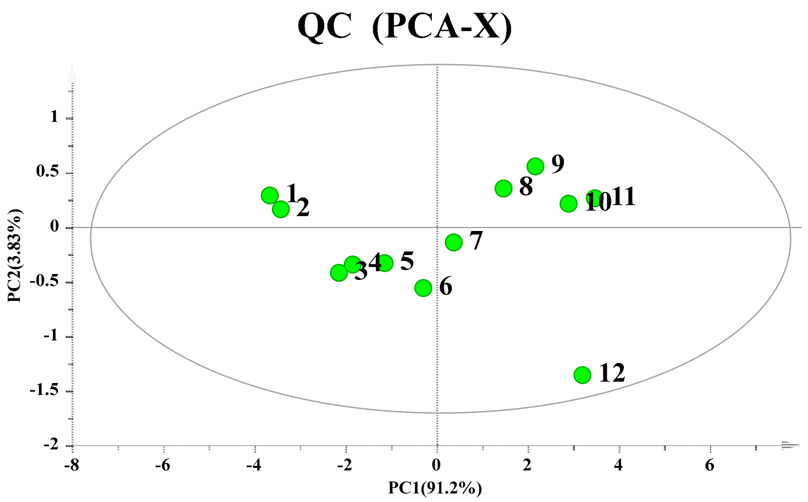

The clustering of 12 QC samples was checked using principal components analysis. The clustering of in-run quality control samples supports the good overall repeatability during the sequence, indicating the robustness of acquired data.

2) Other biomarkers

Measurements of H3Cit, MPO, dsDNA, MCP-1, RANTES, CD163, Lp-PLA2, PAI-1, P-selectin, and D-dimer were assessed by ELASA Kits. For each outcome measure, Levey-Jennings charts were performed for quality control (QC), and Westgard rules were used to assess the QC [27–29].

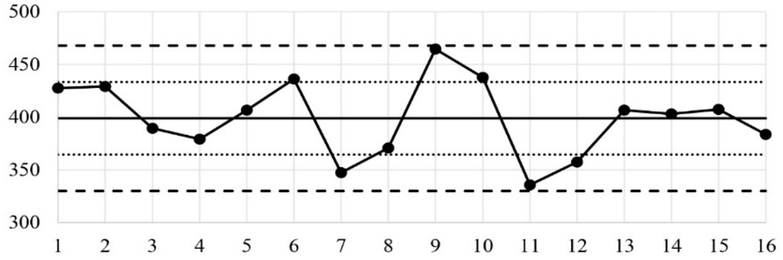

Levey-Jennings chart for MPO

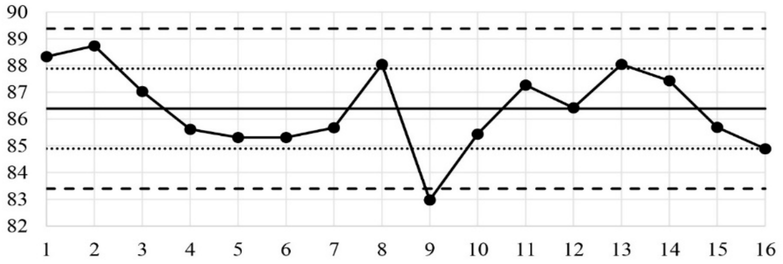

Levey-Jennings chart for MCP-1

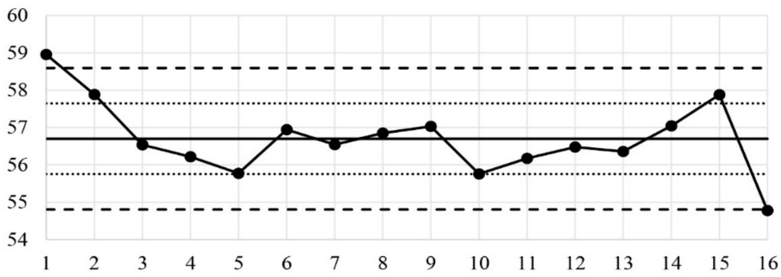

Levey-Jennings chart for RANTES

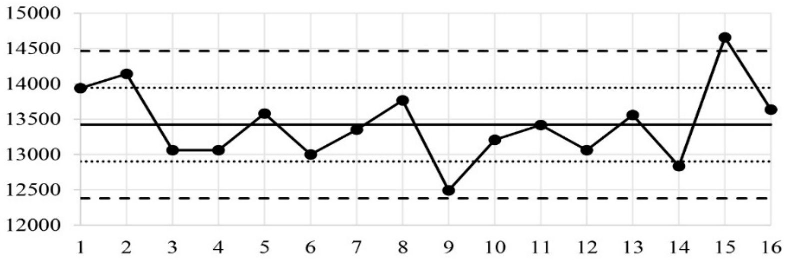

Levey-Jennings chart for CD163

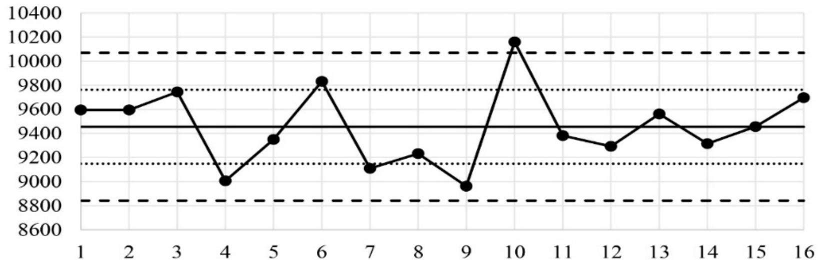

Levey-Jennings chart for LpPLA2

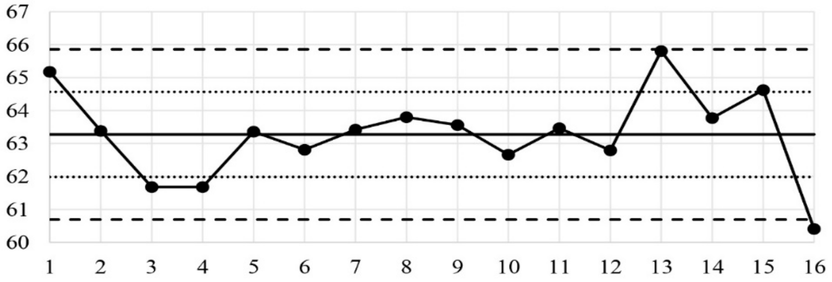

Levey-Jennings chart for PAI-1

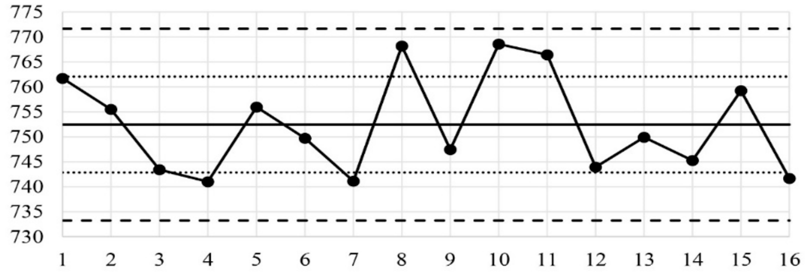

Levey-Jennings chart for P-selectin

All L-J charts were plotted based on standard samples with known concentrations.

—— represents mean; – – · represents mean ± 2SD; ······· represents mean ± SD.

Standard samples in QA data were almost within ± 2SD data which were considered within the "desired precision". Some QA of biomarkers (e.g., MCP-1, RANTES, CD163) performed 1-2S, which represented a "warning sign" and not evidence of an "out of control" condition.

Table S1. Concentrations of ambient $PM_{2.5}$ and its components during the study period.

Pollutants (μg/m^3)	Mean (SD)	Median (IQR)
$PM_{2.5}$	83.32 (53.9)	73.0 (66.9)
$PM_{2.5}$ components		
OA	11.9 (9.1)	10.8 (10.4)
COA	2.1 (1.2)	1.9 (1.8)
FFOA	1.0 (0.9)	0.6 (1.1)
LO-OOA	2.2 (2.3)	1.4 (2.4)
MO-OOA	6.3 (5.7)	5.0 (6.3)
SO_4^{2-}	4.6 (5.8)	2.7 (5.0)
NO_3^-	8.5 (9.2)	5.7 (9.0)
NH_4^+	4.6 (4.4)	3.2 (5.0)
Cl^-	0.3 (0.5)	0.1 (0.2)

Environmental measures were averaged concentration on the full hour of blood draws. Abbreviations: $PM_{2.5}$, particulate matter in diameter <2.5 μm; OA, organic aerosol; COA, cooking organic aerosol; FFOA, fossil fuel-related organic aerosol; LO-OOA, a less oxidized oxygenated organic aerosol; MO-OOA, a more oxidized oxygenates organic aerosol; SO_4^{2-}, sulfate; NO_3^-, nitrate; NH_4^+, ammonium; Cl^-, chloride.

Table S2. Results from single-mediator models.

Pollutants	MA (hours)	Outcomes	Mediators	Mediation effects*	Proportion mediated (%)
NH_4^+	72	RANTES	Cer C16:0	0.0023 (0.0005, 0.0052)	5.28
MO-OOA	72	RANTES	Cer C16:0	0.0029 (0.0003, 0.0063)	3.73
SO_4^{2-}	18	PAI-1	RANTES	0.0038 (0.0002, 0.0074)	8.32
NO_3^-	18	PAI-1	RANTES	0.0016 (0.0001, 0.0031)	5.30
MO-OOA	24	PAI-1	RANTES	0.0061 (0.0015, 0.0117)	8.27
MO-OOA	48	PAI-1	RANTES	0.0046 (0.0009, 0.0092)	5.27

*We assessed possible mediators of the associations between $PM_{2.5}$ components and health outcomes; the statistical significance (p-value <0.05) of the mediation effects was determined by examining whether the 95% confidence interval (CI) contains 0. Averaging time represents concentrations of $PM_{2.5}$ components over the 1 to 72 hours prior to each participant's clinic visit. Abbreviations: MA, moving average; IQR; interquartile range; OA, organic aerosol; COA, cooking organic aerosol; FFOA, fossil fuel-related organic aerosol; LO-OOA, a less oxidized oxygenated organic aerosol; MO-OOA, a more oxidized oxygenates organic aerosol; SO_4^{2-}, sulfate; NO_3^-, nitrate; NH_4^+, ammonium; Cl^-, chloride; H3cit, citrullinated histone H3; dsDNA, double-stranded DNA; MPO, myeloperoxidase; PAI-1, plasminogen activator inhibitor 1.

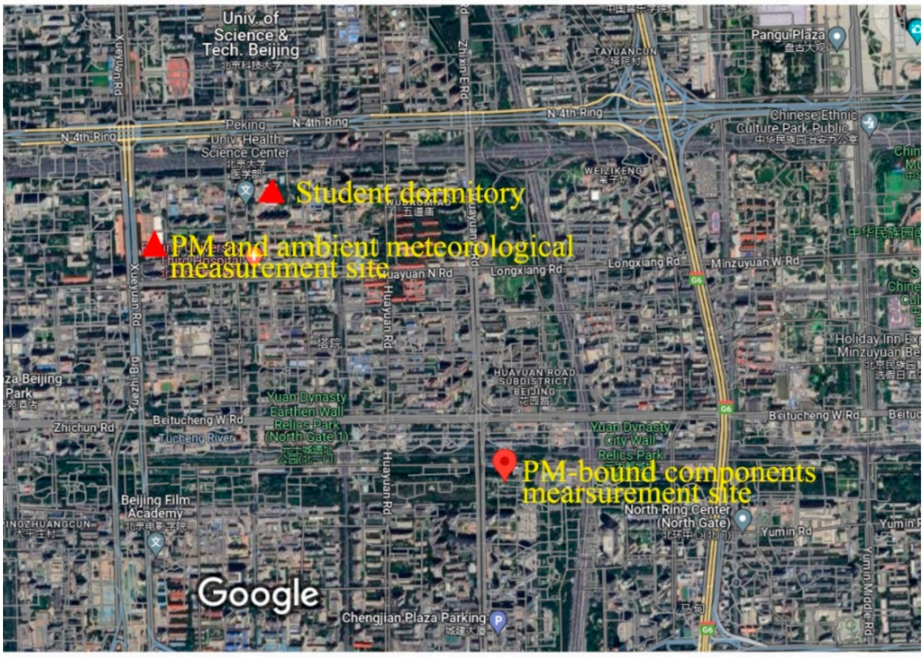

Imagery ©2024 Airbus, CNES / Airbus, Maxar Technologies, Map data ©2024 200 m

Figure S1. Maps of the sampling sites and residential location of participants. ©Google Earth. $PM_{2.5}$ components were measured at the tower branch of Institute of Atmospheric Physics, Chinese Academy of Sciences (39º58′ 28″ N, 116º22′16″ E), which located 1.5 kilometers away from the site in Peking University Health Science Center (39°58′58″N, 116°20′52″ E) measuring total $PM_{2.5}$ concentration and ambient meteorological.

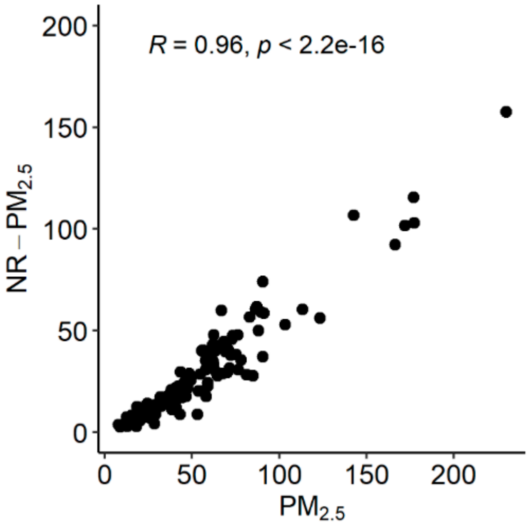

Figure S2. The scatter plot of NR-$PM_{2.5}$ to $PM_{2.5}$ in each year from September, 2019 to January, 2020.
Non-refractory $PM_{2.5}$ (NR-$PM_{2.5}$) was defined as the sum of all components as represent of $PM_{2.5}$ measured at the tower branch site in IPA. $PM_{2.5}$ concentrations in the scatter plot were measured at PUHSC campus.

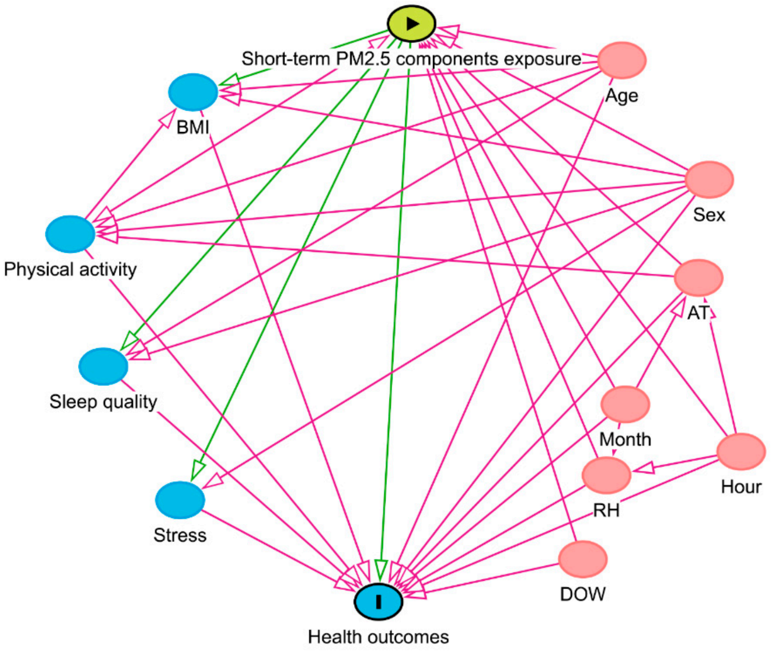

Figure S3. Confounding variables for characterizing the associations between air pollution exposure and health outcomes selected by the directed acyclic graph. Age, sex, month of the blood withdrawal, day of the week and hour of the blood withdrawal, physical activity, ambient temperature, and relative humidity were selected in the main LME models.

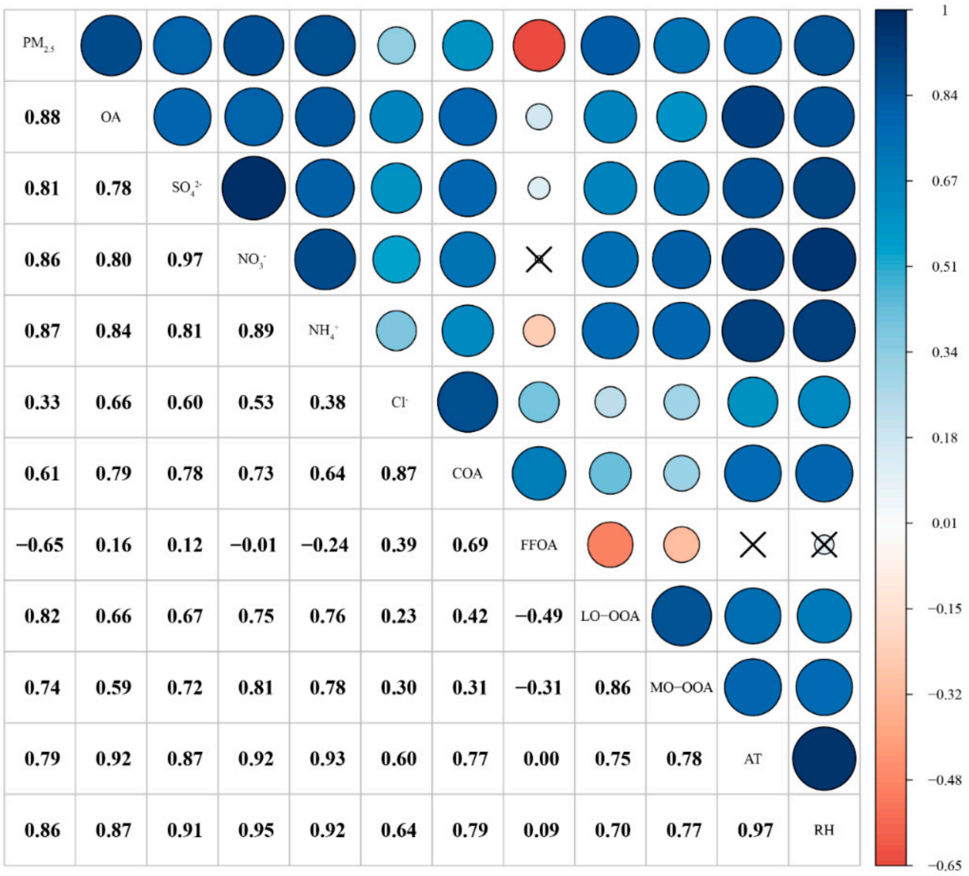

Figure S4. Spearman's correlations between $PM_{2.5}$ components and meteorological parameters. Abbreviations: $PM_{2.5}$, particulate matter in diameter <2.5 μm; OA, organic aerosol; SO_4^{2-}, sulfate; NO_3^-, nitrate (NO_3^-); NH_4^+, ammonium; Cl^-, chloride; COA, cooking organic aerosol; FFOA, fossil fuel-related organic aerosol; LO-OOA, a less oxidized oxygenated organic aerosol; MO-OOA, a more oxidized oxygenates organic aerosol.

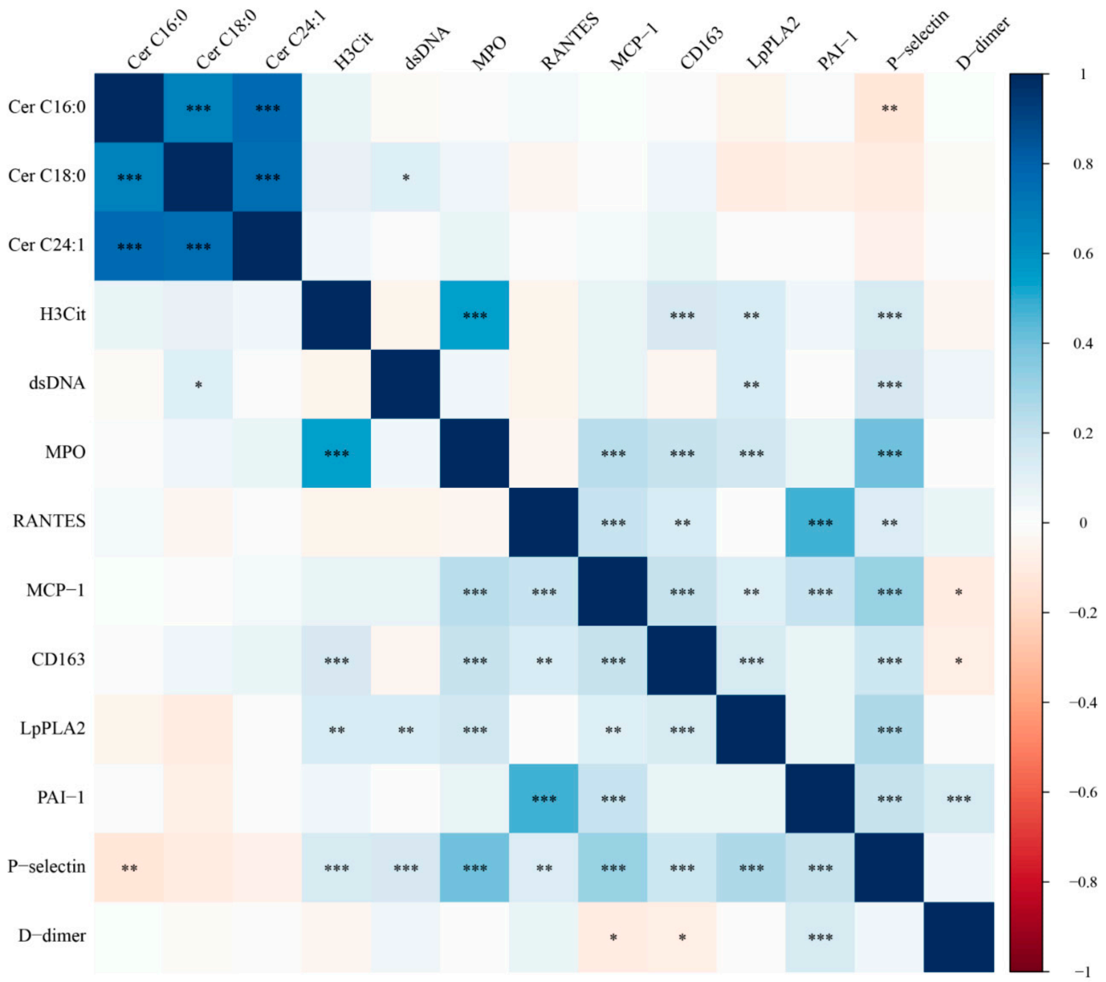

Figure S5. Spearman’s correlations of measured biomarkers. Significant correlation coefficients are indicated by asterisks: * *p*-value <0.05; ** *p*-value <0.01; *** *p*-value <0.001. Abbreviations: H3Cit, citrullinated histone H3; dsDNA, double-stranded DNA; MPO, myeloperoxidase; RANTES, regulated upon activation normal T cell expressed and secreted; MCP-1, monocyte chemoattractant protein-1; sCD163, soluble CD163; Lp-PLA2, lipoprotein-associated phospholipase A2; PAI-1, plasminogen activator inhibitor-1.

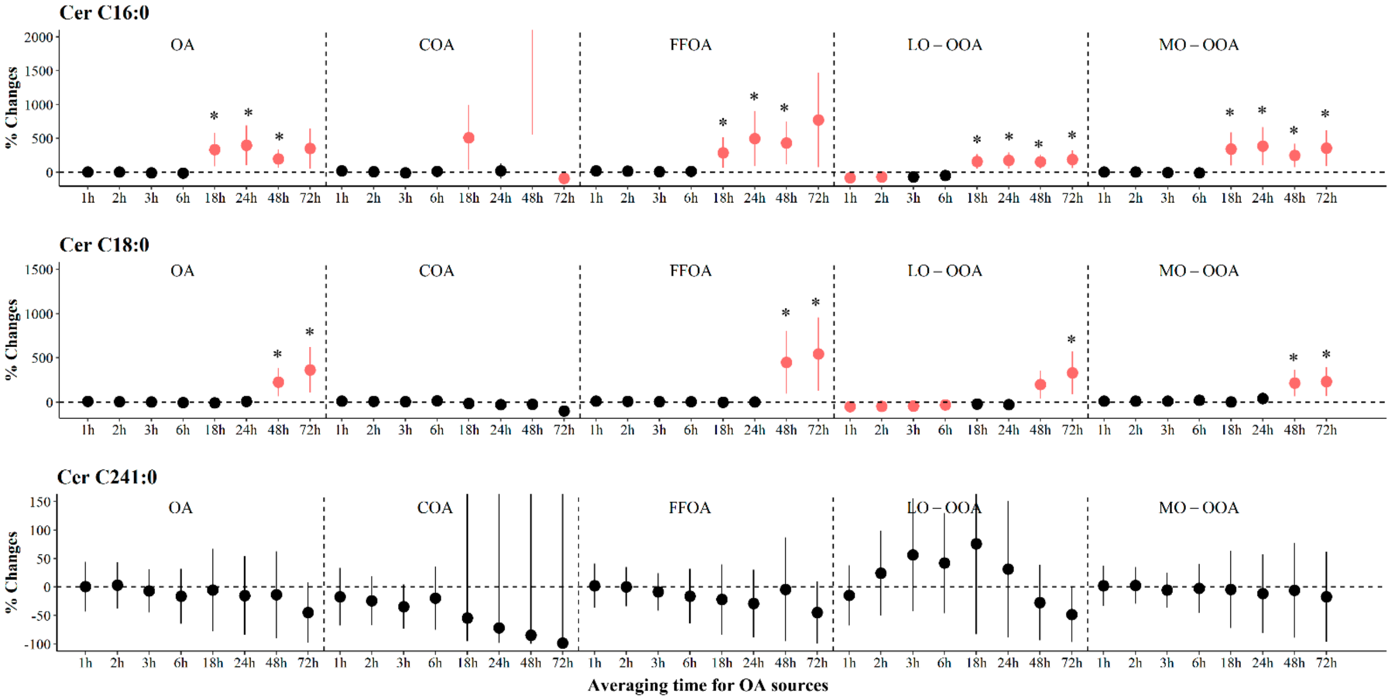

Figure S6. The adjusted percentage changes in ceramides associated with per IQR increment in source-specific OA. Averaging time represents the mean of source-specific OA concentrations over the 1 to 72 hours (3 days) prior to each participant's clinic visit. Error bars indicated 95% CI. Significant association was shown in red (p<0.05) Bonferroni corrections with significance (p<0.0026) are indicated by asterisks. Abbreviations: IQR; interquartile range; Cer, ceramides; OA, organic aerosol; COA, cooking organic aerosol; FFOA, fossil fuel-related organic aerosol; LO-OOA, a less oxidized oxygenated organic aerosol; MO-OOA, a more oxidized oxygenates organic aerosol.

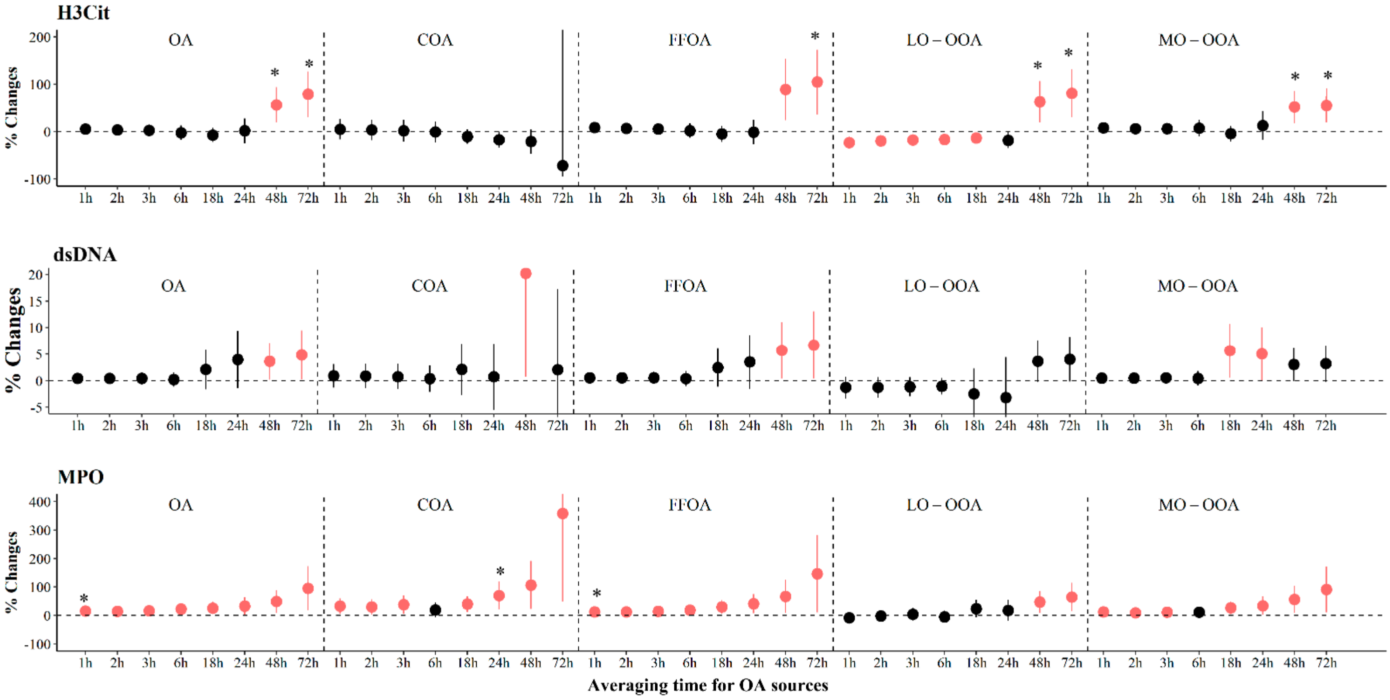

Figure S7. The adjusted percentage changes in NETs associated with per IQR increment in source-specific OA. Averaging time represents the mean of source-specific OA concentrations over the 1 to 72 hours (3 days) prior to each participant's clinic visit. Error bars indicated 95% *CI*. Significant association was shown in red (p<0.05). Bonferroni corrections with significance (p<0.0026) are indicated by asterisks. Abbreviations: IQR; interquartile range; NET, neutrophil extracellular traps; H3Cit, citrullinated histone H3; dsDNA, double-stranded DNA; MPO, myeloperoxidase; OA, organic aerosol; COA, cooking organic aerosol; FFOA, fossil fuel-related organic aerosol; LO-OOA, a less oxidized oxygenated organic aerosol; MO-OOA, a more oxidized oxygenates organic aerosol.

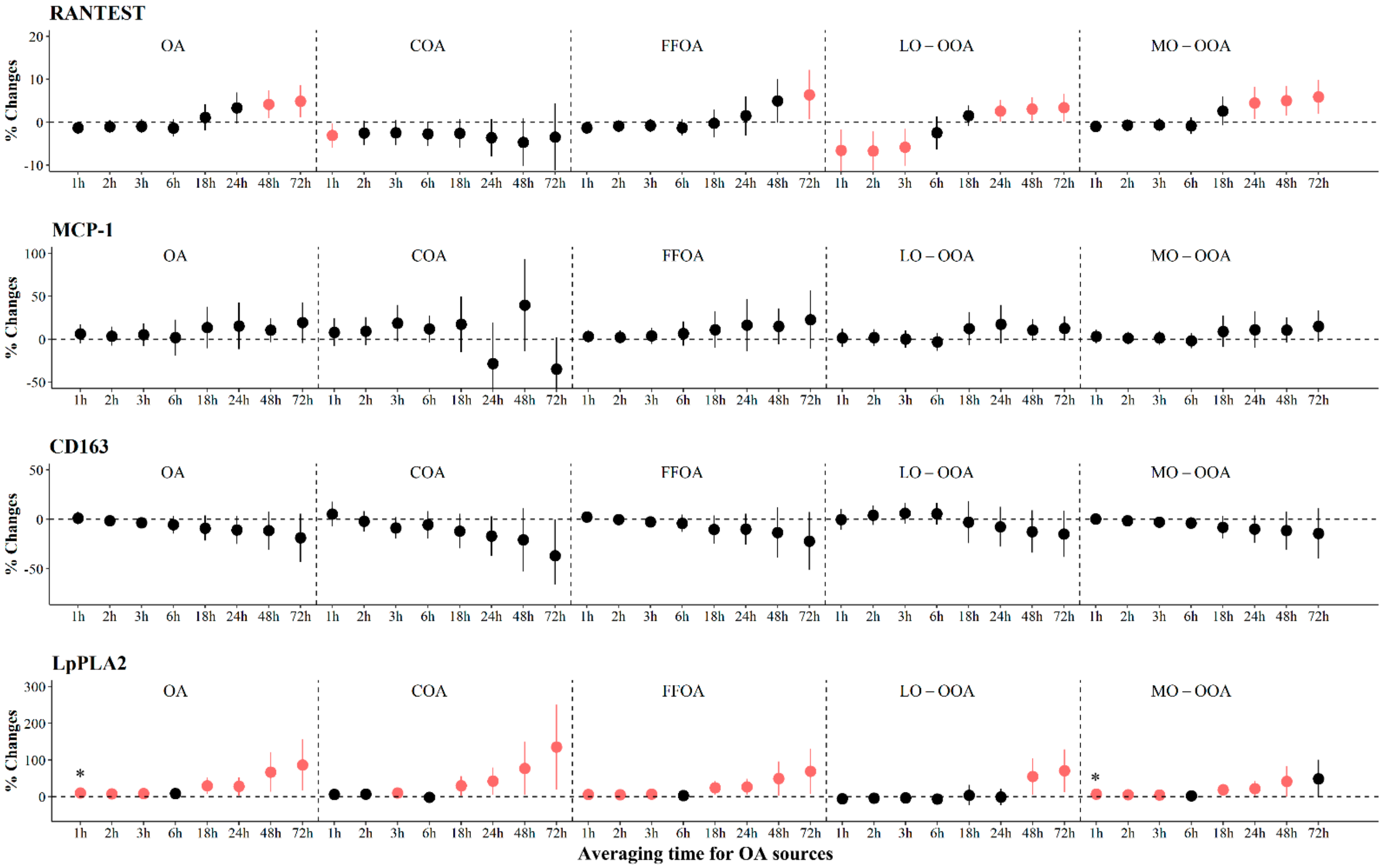

Figurer S8. The adjusted percentage changes in pro-inflammation biomarkers associated with IQR increment in source-specific OA. Averaging time represents the mean of source-specific OA concentrations over the 1 to 72 hours (3 days) prior to each participant's clinic visit. Error bars indicated 95% *CI*. Significant association was shown in red (p<0.05). Bonferroni corrections with significance (p<0.0026) are indicated by asterisks. Abbreviations: IQR; interquartile range; RANTES, regulated upon activation normal T cell expressed and secreted; MCP-1, monocyte chemoattractant protein-1; Lp-PLA2, lipoprotein-associated phospholipase A2; OA, organic aerosol; COA, cooking organic aerosol; FFOA, fossil fuel-related organic aerosol; LO-OOA, a less oxidized oxygenated organic aerosol; MO-OOA, a more oxidized oxygenates organic aerosol.

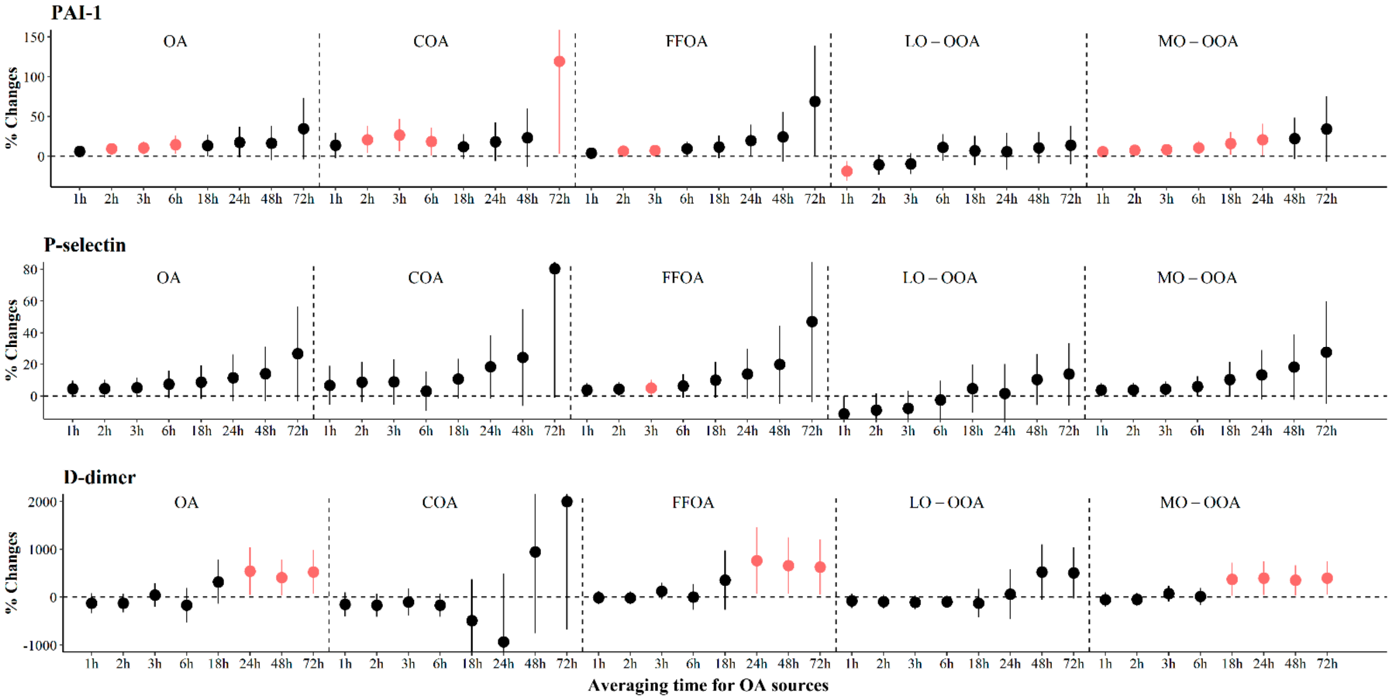

Figure S9. The adjusted percentage changes in coagulation associated with IQR increment in source-specific OA. Averaging time represents the mean of source-specific OA concentrations over the 1 to 72 hours (3 days) prior to each participant's clinic visit. Error bars indicated 95% *CI*. Significant association was shown in red (p<0.05). Bonferroni corrections with significance (p<0.0026) are indicated by asterisks. Abbreviations: IQR; interquartile range; PAI-1, plasminogen activator inhibitor-1; OA, organic aerosol; COA, cooking organic aerosol; FFOA, fossil fuel-related organic aerosol; LO-OOA, a less oxidized oxygenated organic aerosol; MO-OOA, a more oxidized oxygenates organic aerosol.

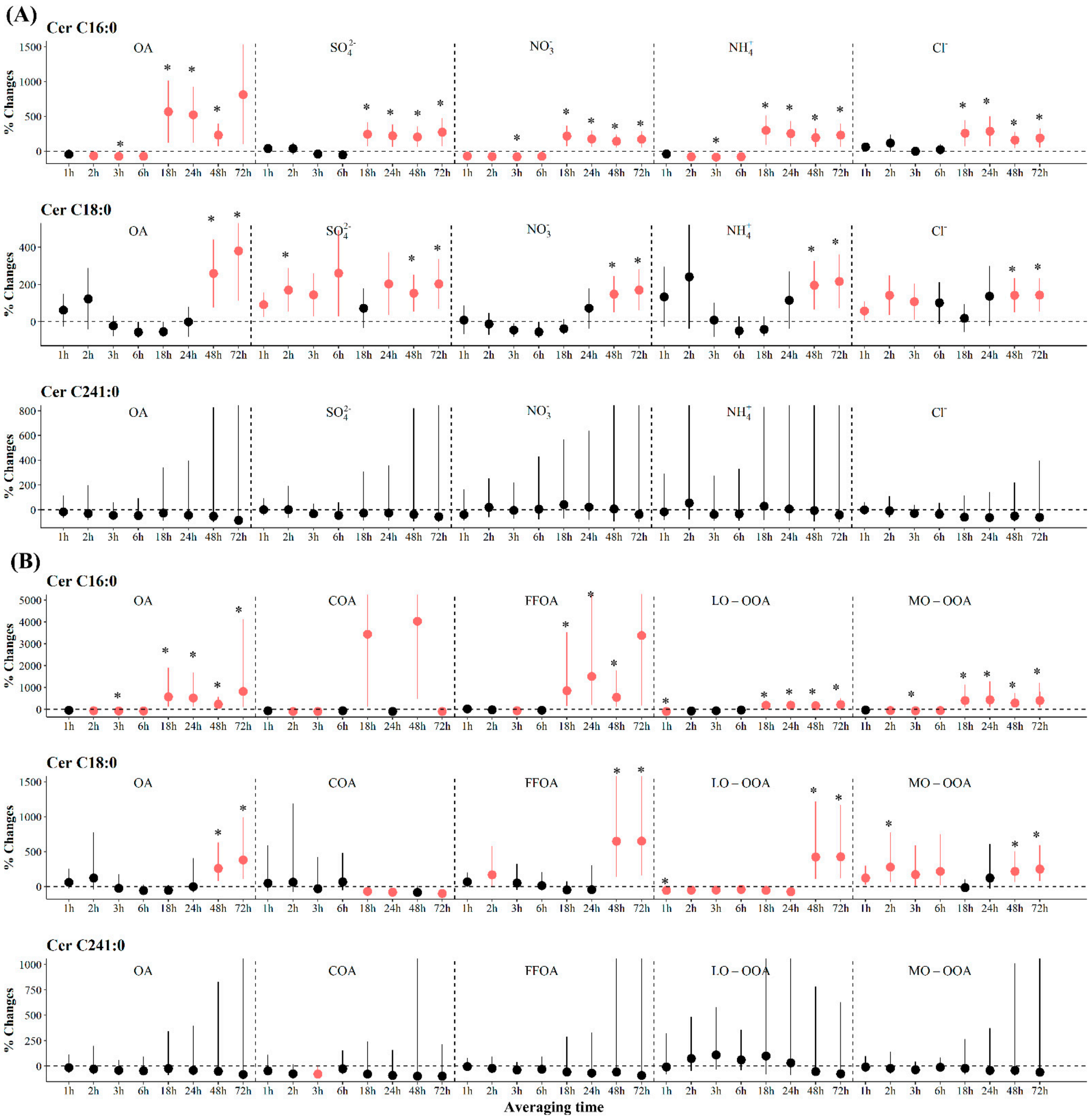

Figure S10. Percentage changes in ceramides associated with $PM_{2.5}$ components (A) and source-specific OA (B) adjusted by $PM_{2.5}$. Averaging time represents the mean of $PM_{2.5}$ component and source-specific OA concentrations over the 1 to 72 hours (3 days) prior to each participant's clinic visit. Error bars indicated 95% *CI*. Significant association was shown in red (p<0.05). Bonferroni corrections with significance (p<0.0026) are indicated by asterisks. Abbreviations: MA, moving average; IQR; interquartile range; Abbreviations: Cer, ceramides; OA, organic aerosol; IA, inorganic aerosol; SO_4^{2-}, sulfate; NO_3^-, nitrate (NO_3^-); NH_4^+, ammonium; Cl^-, chloride.

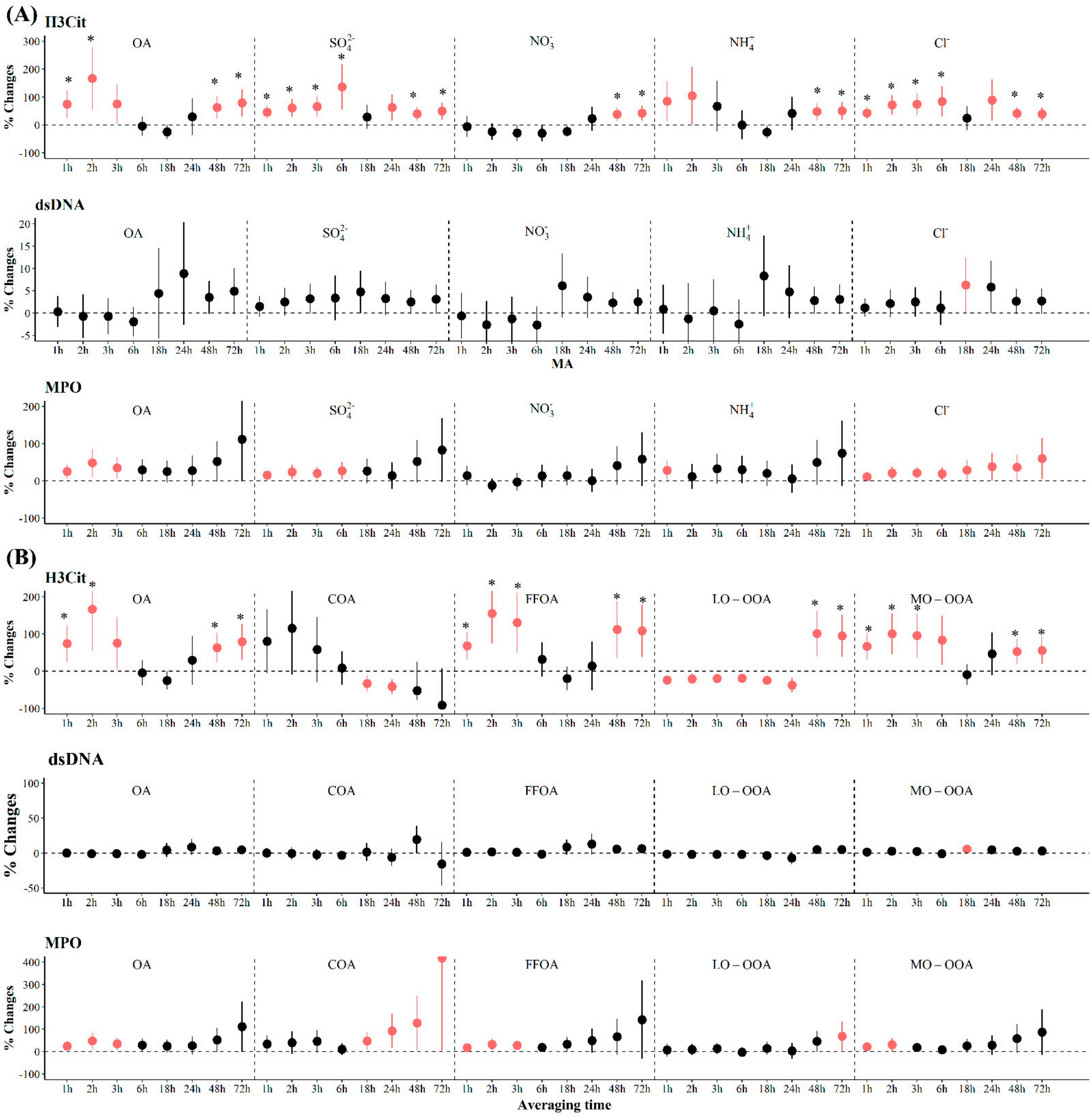

Figure S11. Percentage changes in NETs associated with $PM_{2.5}$ components (A) and source-specific OA (B) adjusted by $PM_{2.5}$. Averaging time represents the mean of $PM_{2.5}$ component and source-specific OA concentrations over the 1 to 72 hours (3 days) prior to each participant's clinic visit. Error bars indicated 95% *CI*. Significant association was shown in red ($p<0.05$). Bonferroni corrections with significance ($p<0.0026$) are indicated by asterisks. Abbreviations: IQR; interquartile range; NET, neutrophil extracellular traps; H3Cit, citrullinated histone H3; dsDNA, double-stranded DNA; MPO, myeloperoxidase; OA, organic aerosol; IA, inorganic aerosol; SO_4^{2-}, sulfate; NO_3^-, nitrate (NO_3^-); NH_4^+, ammonium; Cl^-, chloride.

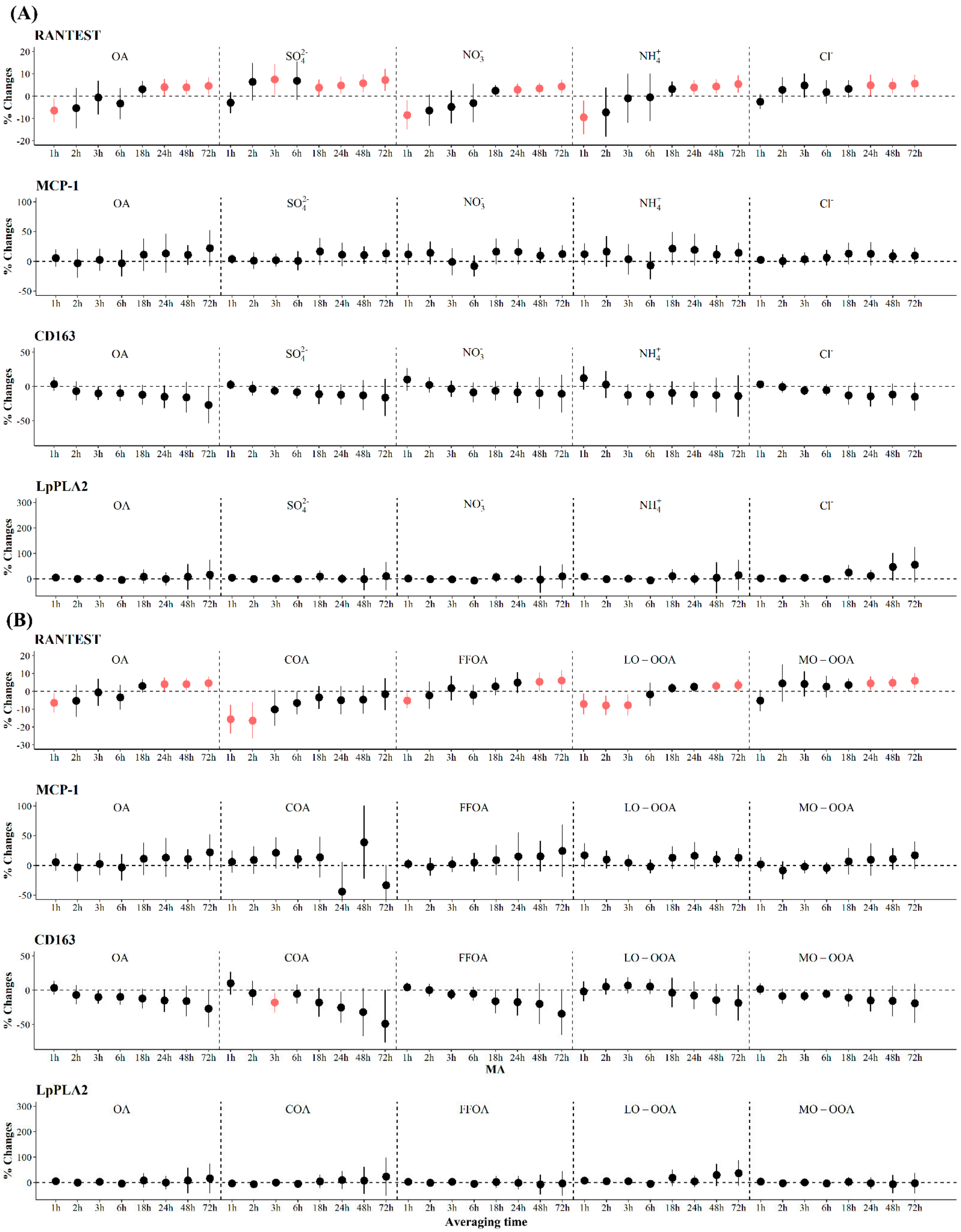

Figure S12. Percentage changes in systemic inflammation associated with $PM_{2.5}$ components (A) and source-specific OA (B) adjusted by $PM_{2.5}$. Averaging time represents the mean of $PM_{2.5}$ component and source-specific OA concentrations over the 1 to 72 hours (3 days) prior to each participant's clinic visit. Error bars indicated 95% *CI*. Significant association was shown in red (p<0.05). Bonferroni corrections with significance (p<0.0026) are indicated by asterisks. Abbreviations: IQR;

interquartile range; RANTES, regulated upon activation normal T cell expressed and secreted; MCP-1, monocyte chemoattractant protein-1; Lp-PLA2, lipoprotein-associated phospholipase A2; OA, organic aerosol; COA, cooking organic aerosol; FFOA, fossil fuel-related organic aerosol; LO-OOA, a less oxidized oxygenated organic aerosol; MO-OOA, a more oxidized oxygenates organic aerosol.

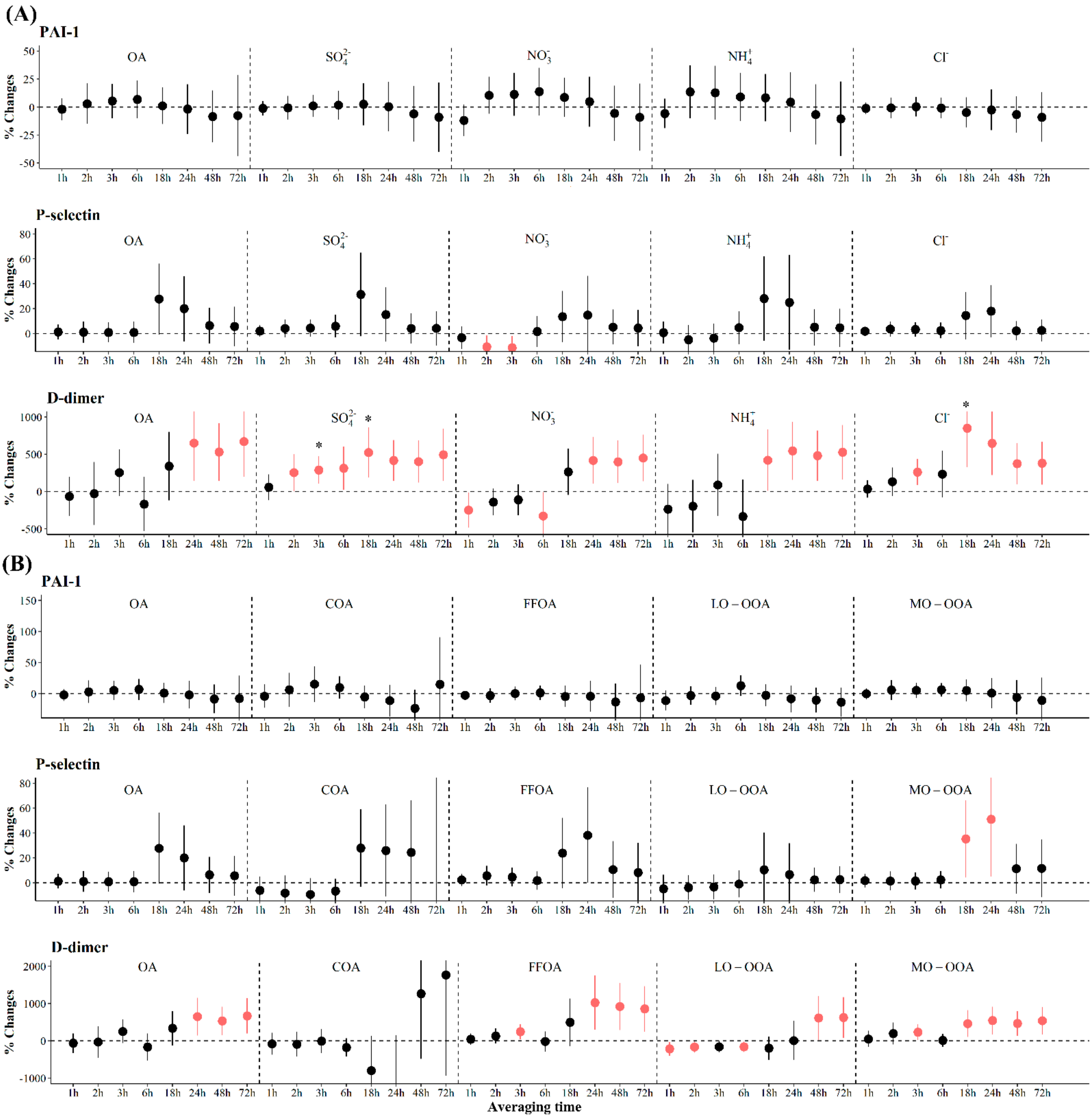

Figure S13. Percentage changes in coagulation associated with $PM_{2.5}$ components (A) and source-specific OA (B) adjusted by $PM_{2.5}$. Averaging time represents the mean of $PM_{2.5}$ component and source-specific OA concentrations over the 1 to 72 hours (3 days) prior to each participant's clinic visit. Error bars indicated 95% *CI*. Significant association was shown in red (p<0.05). Bonferroni corrections with significance (p<0.0026) are indicated by asterisks. Abbreviations: IQR; interquartile range; PAI-1, plasminogen activator inhibitor-1; OA, organic aerosol; IA, inorganic aerosol; SO_4^{2-}, sulfate; NO_3^-, nitrate (NO_3^-); NH_4^+, ammonium; Cl^-, chloride.
